# A MYB4-MAN3-Mannose-MNB1 signaling cascade regulates cadmium tolerance in *Arabidopsis*

**DOI:** 10.1371/journal.pgen.1009636

**Published:** 2021-06-28

**Authors:** Xingxing Yan, Ying Huang, Hui Song, Feng Chen, Qingliu Geng, Min Hu, Cheng Zhang, Xi Wu, Tingting Fan, Shuqing Cao

**Affiliations:** School of Food and Biological Engineering, Hefei University of Technology, Hefei, China; The University of North Carolina at Chapel Hill, UNITED STATES

## Abstract

Our previous studies showed that MAN3-mediated mannose plays an important role in plant responses to cadmium (Cd) stress. However, the underlying mechanisms and signaling pathways involved are poorly understood. In this study, we showed that an *Arabidopsis* MYB4-MAN3-Mannose-MNB1 signaling cascade is involved in the regulation of plant Cd tolerance. Loss-of-function of *MNB1* (mannose-binding-lectin 1) led to decreased Cd accumulation and tolerance, whereas overexpression of *MNB1* significantly enhanced Cd accumulation and tolerance. Consistently, expression of the genes involved in the GSH-dependent phytochelatin (PC) synthesis pathway (such as *GSH1*, *GSH2*, *PCS1*, and *PCS2*) was significantly reduced in the *mnb1* mutants but markedly increased in the *MNB1-OE* lines in the absence or presence of Cd stress, which was positively correlated with Cd-activated PC synthesis. Moreover, we found that mannose is able to bind to the GNA-related domain of MNB1, and that mannose binding to the GNA-related domain of MNB1 is required for MAN3-mediated Cd tolerance in *Arabidopsis*. Further analysis showed that MYB4 directly binds to the promoter of *MAN3* to positively regulate the transcript of *MAN3* and thus Cd tolerance via the GSH-dependent PC synthesis pathway. Consistent with these findings, overexpression of *MAN3* rescued the Cd-sensitive phenotype of the *myb4* mutant but not the *mnb1* mutant, whereas overexpression of *MNB1* rescued the Cd-sensitive phenotype of the *myb4* mutant. Taken together, our results provide compelling evidence that a MYB4-MAN3-Mannose-MNB1 signaling cascade regulates cadmium tolerance in *Arabidopsis* through the GSH-dependent PC synthesis pathway.

## Author summary

Cadmium (Cd) pollution in soils is recognized as an environmental problem worldwide, and phytoremediation is one of the important approaches for cleaning Cd-contaminated soils. However, the molecular mechanisms involved in Cd tolerance remains unclear. Here we demonstrated that overexpression of *MNB1*, which encodes a mannose-binding lectin, manifestly increased Cd tolerance, whereas loss-of-function of *MNB1* led to enhanced Cd sensitivity. Further analysis showed that mannose binding to the GNA-related domain of MNB1 is required for MAN3-mediated Cd tolerance. Moreover, under Cd stress, MYB4 directly binds the promoter of *MAN3* to positively regulate the expression of *MAN3*, and thus Cd tolerance via the glutathione (GSH)-dependent phytochelatin (PC) synthesis pathway. Our results demonstrated that a MYB4-MAN3-Mannose-MNB1 signaling cascade regulates Cd tolerance through the GSH-dependent PC synthesis pathway in *Arabidopsis*.

## Introduction

The heavy metal cadmium (Cd) is a toxic trace pollutant for humans, animals and plants, which enters the environment mainly from the burning of fossil fuels and phosphate fertilizers and thus the food chain [1–3]. Studies have revealed that Cd can denature proteins by binding to sulphydryl groups and induce oxidative stress, and thereby causes cellular damage by displacing cofactors from a variety of proteins, such as enzymes and transcription factors [4–7].

Plants have evolved various efficient mechanisms for Cd detoxification and tolerance, and these mechanisms include Cd chelation, control of Cd influx, scavenging of Cd-induced reactive oxygen species, extrusion of Cd across the plasma membrane, Cd sequestration and remobilization, and sequestration of Cd into vacuoles [6–7]. In addition, the antioxidative defence system and signaling mechanisms may also participate in the process [2,8–11]. More recently, a number of key genes have been shown to be involved in regulation of Cd detoxification and tolerance in different plant species, such as ZNT1, GSH1, OsHMA9, AtPDR8, ZntA, CAD2, ATM3, Nramp5, and ACBP1 [4,7,12–20]. There is convincing evidence that the products encoded by a large proportion of these genes are chelators [21,22]. Recent studies demonstrate that transcription factors are critical components in regulating Cd detoxification and tolerance in plants, such as HsfA4a [23], bHLH29, bHLH38, and bHLH39 [24], ZAT6 [25], OXIDATIVE STRESS2 [26], and MYB49 [27]. MYB4 was shown to be a MYB transcription factor involved in regulating flavonoid biosynthesis [28], the UV-B response [29] and the defence response [30]. However, whether and how MYB4 regulates the plant response to Cd stress remains unknown.

It is well known that carbohydrate-binding proteins are commonly referred to as lectins, which are ubiquitous in many plants [31]. Plant mannose-binding lectins are crucial for plant defense signaling during pathogen attack by recognizing specific carbohydrates on pathogen surfaces [32]. All known plant lectins can be divided into 12 lectin families of structurally and evolutionarily related proteins [33]. Among them, monocot mannose-binding and cucurbitaceae phloem lectins were now called the GNA-related and the *Nictaba* lectins, respectively [34]. It was shown that the pepper mannose-binding lectin CaMBL1 is involved in defense against microbial pathogens [32], and mannose has been observed to bind to plant lectins that possess diverse biological roles [34–36]. Interestingly, we found that MAN3-mediated mannose plays a vital role in plant response to Cd stress [37]. Thus, whether the *Arabidopsis* homolog of CaMBL1 (mannose-binding-lectin, MNB1) is involved in the regulation of MAN3-mediated Cd tolerance remains to be investigated.

In this study, we show that MNB1 plays an vital role in regulating plant response to Cd stress. We demonstrated that overexpression of *MNB1* manifestly increased Cd tolerance, whereas loss-of-function of *MNB1* led to enhanced Cd sensitivity. Further analysis showed that mannose binding to the GNA-related domain of MNB1 is required for MAN3-mediated Cd tolerance. In addition, we also found that, under Cd stress, MYB4 directly binds the promoter of *MAN3* to positively regulate the expression of *MAN3*, and thus Cd tolerance via the glutathione (GSH)-dependent phytochelatin (PC) synthesis pathway. Our results demonstrated that MYB4-MAN3-Mannose-MNB1 signaling cascade regulates Cd tolerance through the GSH-dependent PC synthesis pathway in *Arabidopsis*.

## Results

### *MNB1* is involved in regulating MAN3-mediated Cd tolerance

**Fig 1:**
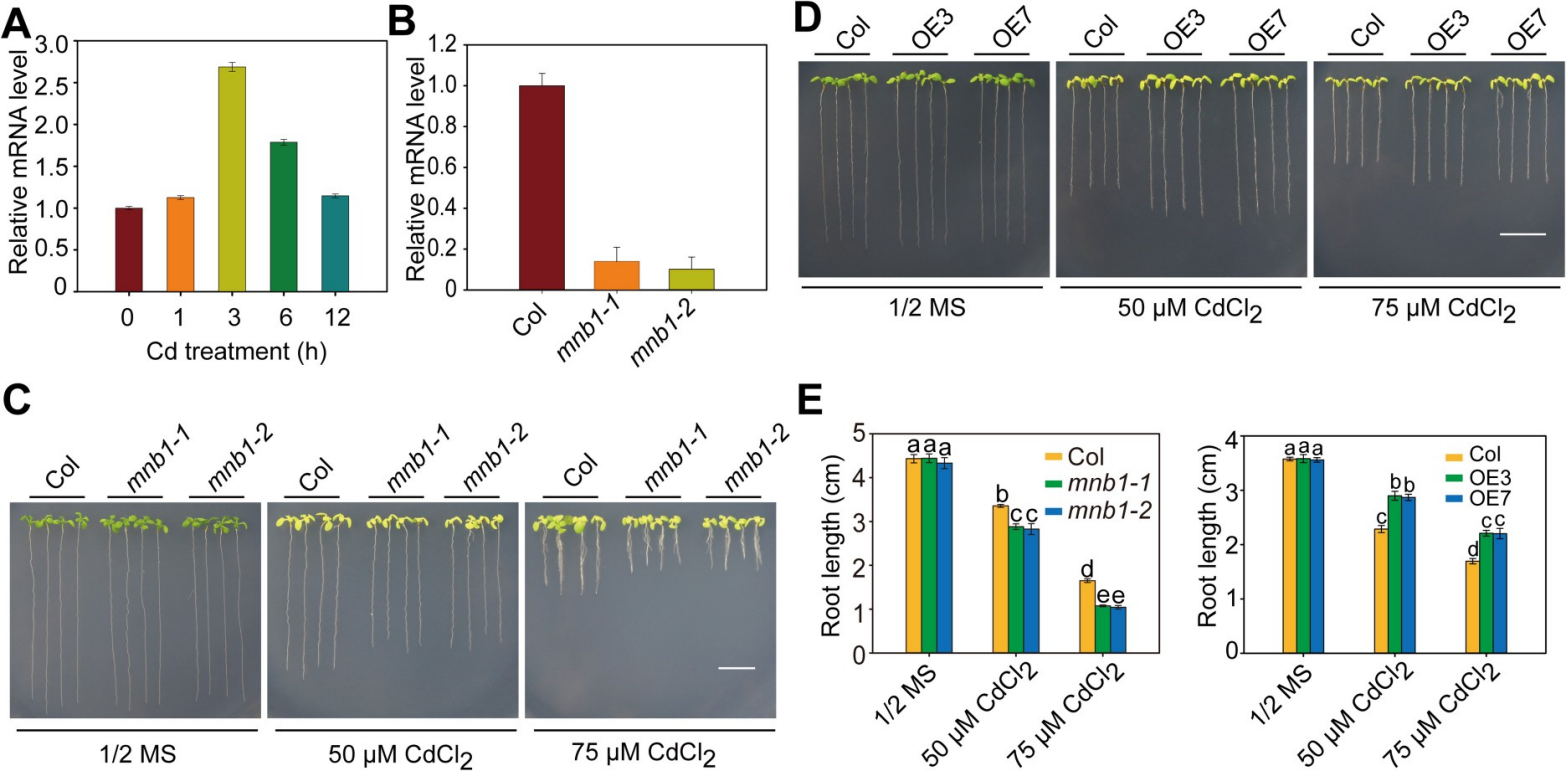
*MNB1* positively regulates Cd tolerance. (A) Expression of *MNB1* in response to Cd stress. Two-week-old wild-type plants grown on 1/2 MS media were treated with 50 μM CdCl_2_ for 0, 1, 3, 6, 12 h before the tissues were harvested for RT-qPCR analysis. *GAPDH* was used as an internal control. Data are presented as means ±SD of three biological replicates. (B) RT-qPCR analysis of *MNB1* transcript level in Col and *mnb1* mutants. *GAPDH* was used as an internal control. Data are presented as means ± SD of three biological replicates. (C, D) Cd stress phenotypes of Col, *mnb1* mutants (C), *MNB1-OE* (D) seedlings. Three-day-old seedlings grown on 1/2 MS medium were transferred to 1/2 MS medium with or without 50 or 75 μM CdCl_2_ for 2 weeks. Scale bar = 1 cm. (E) Root length of plants described in C and D. Three independent experiments were done with similar results, each with three biological replicates. Four plants per genotype from one plate were measured for each replicate. Data are presented as means ± SD, n  =  3. Bars with different lowercase letters are significantly different at *P* < 0.05 (Tukey’s test).

It was shown that MAN3-mediated mannose is involved in the regulation of Cd stress response in *Arabidopsis* [37], and the pepper mannose-binding lectin CaMBL1 has been shown to be involved in defense against microbial pathogens [32]. Therefore, we speculated that the homologous mannose-binding protein may regulate plant responses to Cd stress in *Arabidopsis*. It has been shown that MNB1 (At1g78830) is a member of mannose-binding lectin family, which is the *Arabidopsis* homolog of CaMBL1 [32]. The *MNB1* gene contains one exon, and encodes a 455–amino acid protein with the GNA-related lectin domain (https://www.arabidopsis.org/servlets/TairObject?id=31114&type=locus; [32]). BLAST analysis of *MNB1* in the Nr (non-redundant) protein database of NCBI revealed that it shares >92% sequence similarity with other proteins such as a predicted epidermis-specific secreted glycoprotein EP1-like (*Camelina sativa*) protein (accession no.XP_010429723.1), a predicted epidermis-specific secreted glycoprotein EP1-like (Raphanus sativus) protein (accession no.XP_018465133.1), the hypothetical (*Arabis alpina*) protein (AALP_AA2G232300; accession no.KFK42261.1) and a predicted epidermis-specific secreted glycoprotein EP1-like (*Brassica rapa*) protein (accession no.XP_009106596.1; S1A and S1B Fig). The transcript of *MNB1* was detected in all tissues examined, with higher levels in rosette leaf and root (S1C Fig). In addition, we found that the *MNB1* transcript is induced by Cd stress, reaching to the maximum level after 3 hours of Cd treatment and gradually decreasing afterwards (Fig 1A), suggesting that *MNB1* may be involved in regulating Cd tolerance in *Arabidopsis*.

To test this hypothesis, we characterized two transfer (T)-DNA insertion mutants (*mnb1-1*, SALK_038821C; *mnb1-2*, SALK_121641) for *MNB1* from the SALK T-DNA collection [32,38]; Figs 1B and S1D). These mutants showed similar phenotypes to the growth and development of the Col (S2A Fig). However, under 50 or 75 μM Cd stress, the *mnb1* mutants showed decreased Cd tolerance compared with the Col (Fig 1C). Consistent with this result, root length was significantly shorter in the *mnb1* mutants than in the Col (Fig 1E). These results suggest that *MNB1* is required for Cd tolerance in *Arabidopsis*. In addition, we also tested the responses of the *mnb1* mutants to other stresses, such as Pb(NO_3_)_2,_ ZnSO_4_, CuSO_4_, and Na_3_AsO_4_, and found that *mnb1* mutants showed enhanced sensitive to Cu and Pb but not to As and Zn (S3 Fig).

To further characterize the role of MNB1 in Cd tolerance, we obtained more than eight transgenic *Arabidopsis* lines constitutively expressing MNB1 under the control of the cauliflower mosaic virus (CaMV) 35S promoter, and these lines displayed similar phenotypes to the Col in terms of overall development and flowering time (S4A and S4B Fig). We further chose the OE3 and OE7 lines to analyze their Cd tolerance, and found that the transgenic OE3 and OE7 plants displayed a significantly higher Cd tolerance than wild-type plants (Fig 1D), which is further confirmed by analysis of root length (Fig 1E). Taken together, all these results suggest that *MNB1* positively regulates Cd tolerance in *Arabidopsis*.

### *MNB1* positively regulates Cd tolerance via the GSH-dependent PC synthesis pathway

**Fig 2:**
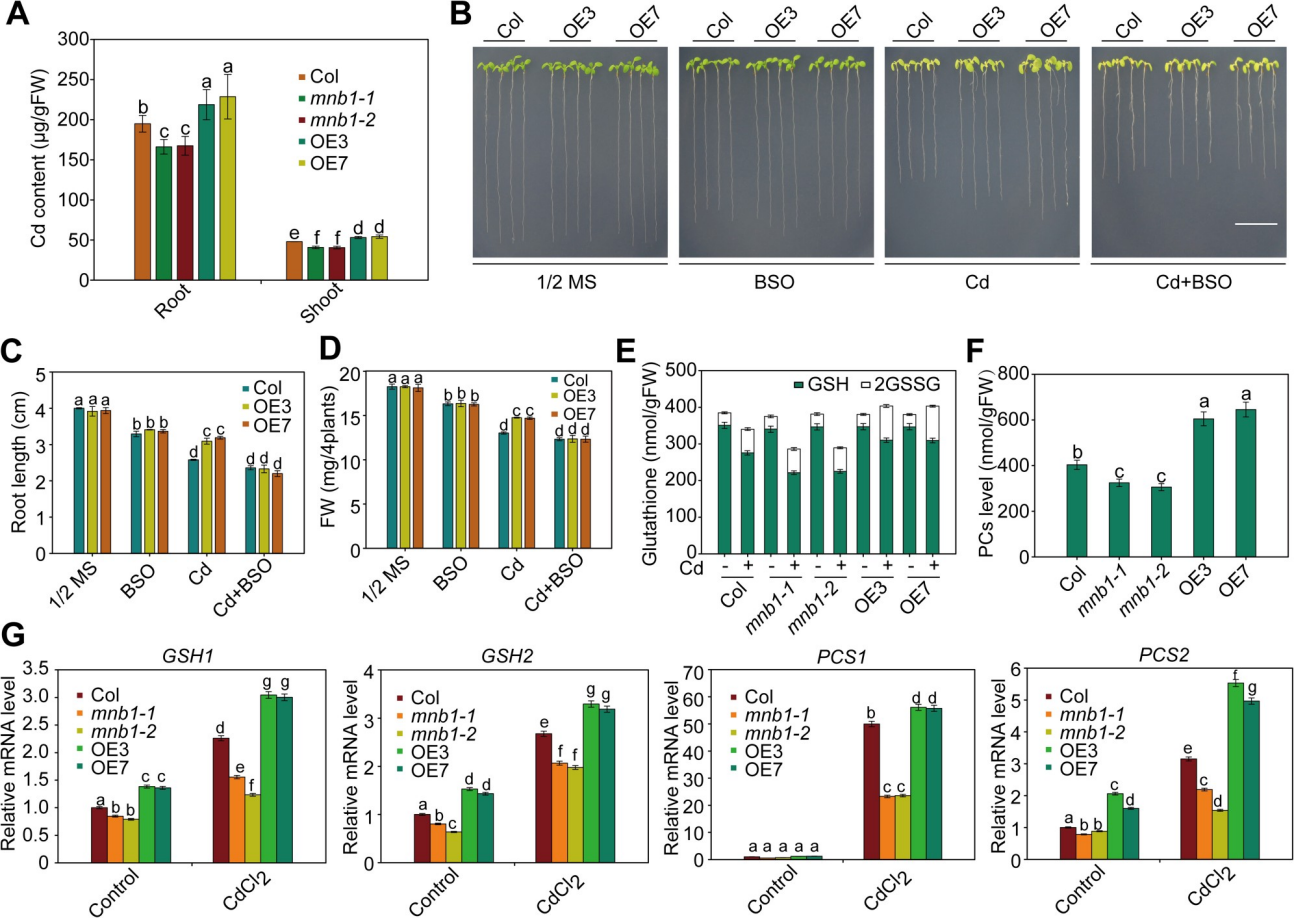
*MNB1* gene positively regulates Cd tolerance by activating expression of PC synthesis-related genes. (A) Cd contents in roots and shoots of Col, *mnb1* mutants, *MNB1-OE* lines. Plants were grown on 1/2 MS medium with 50 μM CdCl_2_ for 2 weeks for analysis of Cd content. Data are presented as means ± SD, n  =  3. Bars with different lowercase letters are significantly different at P < 0.05 (Tukey’s test). (B) Effects of BSO in Col and *MNB1-OE* lines under Cd stress. Three-day-old plants grown on 1/2 MS medium were transferred to 1/2 MS medium with or without 50 μM CdCl_2_ and 0.1 mM BSO for 2 weeks. Scale bar = 1cm. (C, D) Root length (C) and fresh weight (D) of plants described in (B). Three independent experiments were done with similar results, each with three biological repeats. Four plants per genotype from one plate were measured for each repeat. Data are presented as means ±SD, n  =  3. Bars with different lowercase letters are significantly different at *P* < 0.05 (Tukey’s test). (E, F) Total GSH content and total PC content in Col, *mnb1* mutants and *MNB1-OE* plants in the absence or presence of Cd. Two-week-old plants grown on 1/2 MS medium were treated with or without 50 μM CdCl_2_ for 24 h for analysis of glutathione and PC contents. Data are presented as means ± SD, n  =  3. Bars with different lowercase letters are significantly different at P < 0.05 (Tukey’s test). (G) Transcript levels of genes involved in the PC synthesis pathway in Col, *mnb1* mutants and *MNB1-OE* plants in the absence or presence of Cd. *GAPDH* was used as an internal control. Two-week-old plants grown on 1/2 MS medium were treated with or without 50 μM CdCl_2_ for 6 h. Data are presented as means ± SD, n  =  3. Bars with different lowercase letters are significantly different at *P* < 0.05 (Tukey’s test).

It was previously shown that mannose regulates Cd tolerance via the GSH-depenent PC synthesis pathway [37]. Thus, we determine whether *MNB1* also regulates Cd tolerance through this pathway. First, we analyzed Cd content in the Col, *mnb1* mutants, and *MNB1-OE* plants under Cd stress. Cd contents were lower in roots and shoots of *mbn1* mutant plants than in those of the Col; whereas higher Cd contents were detected in the *MNB1-OE* lines (Fig 2A). These results suggest that *MNB1*-mediated Cd tolerance may be associated with a sequestration mechanism. Next, we test whether *MNB1*-mediated Cd tolerance is related to the GSH-dependent PC synthesis pathway [17,39–49]. A GSH synthesis inhibitor buthionine sulfoximine (BSO) was employed to treat the Col and *MNB1*-overexpressing plants. There were no significant differences between Col and MNB1-overexpressing plants grown on medium containing BSO alone; however, Cd tolerance was enhanced in *MNB1*-overexpressing plants than in the Col plants in the medium containing CdCl_2_ (Fig 2B). When BSO was added together with CdCl_2_, the Cd-tolerant phenotype in the *MNB1*-overexpressing plants disappeared (Fig 2B), which is further confirmed by analysis of root length (Fig 2C) and fresh weight (Fig 2D), suggesting that the mechanism of MNB1-mediated Cd tolerance is related to the GSH-dependent PC synthesis pathway. We further measured GSH and PCs levels between the Col and the *mnb1* mutants or *MNB1*-*OE* lines under Cd stress, and found that there were significant differences in GSH (Fig 2E) and PCs (Fig 2F) levels between them under Cd stress, suggesting that a GSH-dependent PC synthesis pathway is indeed involved in the mechanism of MNB1-meidated Cd tolerance. Finally, we analyzed the transcript levels of genes involved in the GSH-dependent PC synthesis pathway in the Col, *mnb1* mutant and *MNB1-OE* plants in response to Cd stress. Expression of the genes involved in the GSH-dependent PC synthesis pathway (such as *GSH1*, *GSH2*, *PCS1*, and *PCS2*) was significantly lower in the *mnb1* mutants, but was markedly higher in the OE lines than in the Col in the absence or presence of Cd stress (Fig 2G). In addition, we also analyzed expression of the other Cd stress-related genes in the Col, *mnb1* mutant and *MNB1-OE* plants in response to Cd stress, and found that *mnb1* mutations affect their expression to different extent (S5 Fig). Taken together, all these results suggest that *MNB1* positively regulates Cd tolerance by mainly activating expression of PC synthesis-related genes.

### Mannose binding to the GNA-related domain of MNB1 is required for MAN3-mediated Cd tolerance

**Fig 3:**
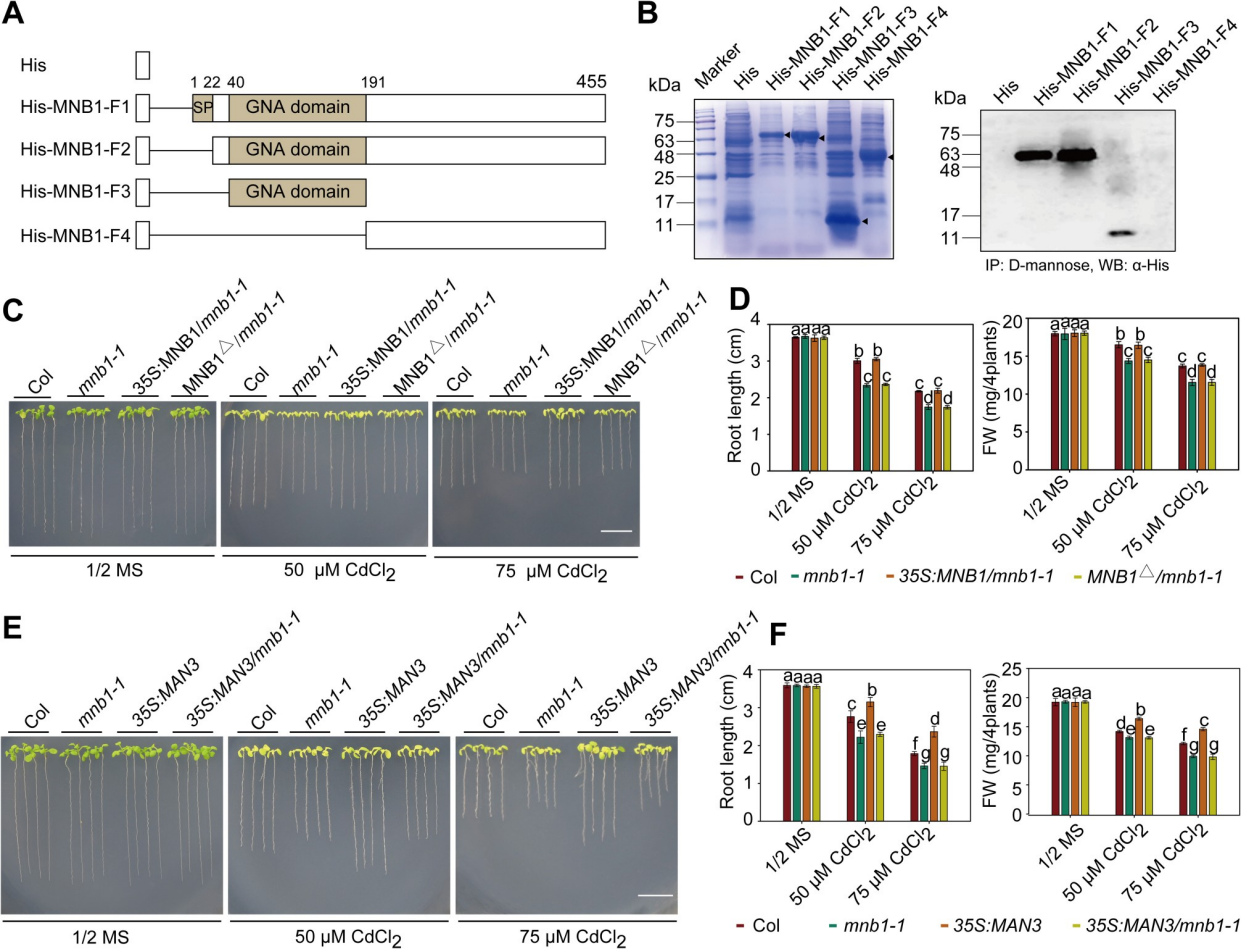
Mannose binding to MNB1 is required for MAN3-mediated Cd tolerance. (A) Schematic representation of MNB1 and MNB1 key deletion constructs. (B) Direct proof of mannose binding to the specific region of MNB1. Immunoblot analysis of MNB1 using the anti-His antibody. His-tagged MNB1 and MNB1 deletion constructs were expressed in *E*. *coli* (left panel) and purified using d-Mannose-agarose columns. Arrowheads indicate expressed His-tagged recombinant proteins. α-His, anti-His antibody; IP, immunoprecipitation; WB, western blotting. (C) Cd tolerance of the Col, *mnb1-1*, *35SMNB1*:*mnb1* and *MNB1*^Δ^/*mnb1* seedlings. (D) Root length and fresh weight of plants described in (C). (E) Cd tolerance of the Col, *mnb1*, *MAN3-OE*, *MAN3-OE/mnbl1* seedlings. (F) Root length and fresh weight of plants described in (E). In (C) and (E), three-day-old seedlings grown on 1/2 MS medium were transferred to 1/2 MS medium with or without 50 or 75 μM CdCl_2_ for about 2 weeks. Scale bar = 1 cm. In (D) and (F), three independent experiments were done with similar results, each with three biological replicates. Four plants per genotype from one plate were measured for each replicate. Data are presented as means ± SD, n  =  3. Bars with different lowercase letters are significantly different at *P* < 0.05 (Tukey’s test).

It was previously shown that CaMBL1 is localized to plasma membrane, and is able to bind to D-Mannose [32]. Consistent with this result, we also found that MNB1 is localized at plasma membrane (S1E Fig).The cell walls have previously been proposed as a major storage location for metals, and the loss of a cell wall protein might cause pleiotropic effects [50,51]. We performed a cell wall structure observation of Col and *mnb1* mutant by scanning electron microscopy (SEM), and found that the cell walls are not seriously affected in the *mnb1* mutant (S1F Fig). To test whether MNB1 is also able to bind to D-Mannose, we created a series of MNB1 deletion mutants based on deduced amino acid sequences of MNB1 (Figs 3A and S6). As shown in Fig 3B, D-mannose is able to bind to the GNA-related domain of MNB1, and the GNA-related domain of MNB1 is essential for its binding to D-mannose.

To further investigate whether the GNA-related domain of MNB1 plays an important role in Cd tolerance, we introduced *35S*:*MNB1* and *35S*:*MNB1*^Δ^ (Deleted section of the GNA-related domain) constructs into the *mnb1-1* mutant to complement its reduced Cd-tolerant phenotypes, respectively (S7A Fig). The resulting *35S*:*MNB1/mnb1-1* plants rescued to the wild-type Cd-tolerant phenotype, but *35S*:*MNB1*^Δ^/*mnb1-1* plants showed similar reduced Cd-tolerant phenotype as *mnb1-1* plants (Fig 3C), as indicated by root length and fresh weight (Fig 3D). These results suggest that the GNA-related domain of MNB1 is required for Cd tolerance.

Based on the fact that MNB1 is able to bind to D-Mannose, we next investigated whether the GNA-related domain of MNB1 is essential for mannose-mediated Cd tolerance. We examined effect of mannose treatment on Cd tolerance of Col, *mnb1-1*, *35S*:*MNB1/mnb1-1* and *35S*:*MNB1*^Δ^/*mnb1-1* seedlings with or without 50 μM CdCl_2_, and found that mannose treatment enhanced Cd tolerance in *35S*:*MNB1/mnb1-1* seedlings but not *35S*:*MNB1*^Δ^/*mnb1-1* seedlings (S8A–S8C Fig), suggesting that the GNA-related domain of MNB1 is required for mannose-mediated Cd tolerance.

To further determine whether the GNA-related domain of MNB1 is required for MAN3-mediated Cd tolerance, we introduced *35S*:*MAN3* construct into the *mnb1-1* mutant, *35S*:*MNB1*/*mnb1-1*, and *35S*:*MNB1*^Δ^/*mnb1-1* lines to test their Cd-tolerant phenotypes (S7B Fig), respectively. The resulting *35S*:*MAN3/mnb1* plants displayed similar decreased Cd-tolerant phenotype as *mnb1* plants (Fig 3E), as indicated by root length and fresh weight (Fig 3F), suggesting that *MAN3* acts upstream of *MNB1* to regulate plant Cd tolerance. Importantly, however, *35S*:*MAN3/35S*:*MNB1*^*Δ*^*/mnb1* plants showed similar reduced Cd-tolerant phenotype as *mnb1-1* plants, as indicated by root length, whereas *35S*:*MAN3/35S*:*MNB1*:*mnb1-1* plants showed similar increased Cd-tolerant phenotype as *35S*:*MAN3* plants (S9 Fig), suggesting that the GNA-related domain of MNB1 is essential for MAN3-meitated Cd tolerance. Collectively, these results indicate that mannose binding to the GNA-related domain of MNB1 is required for MAN3-mediated Cd tolerance.

### MYB4 regulates expression of *MAN3* by directly binding to its promoter

**Fig 4:**
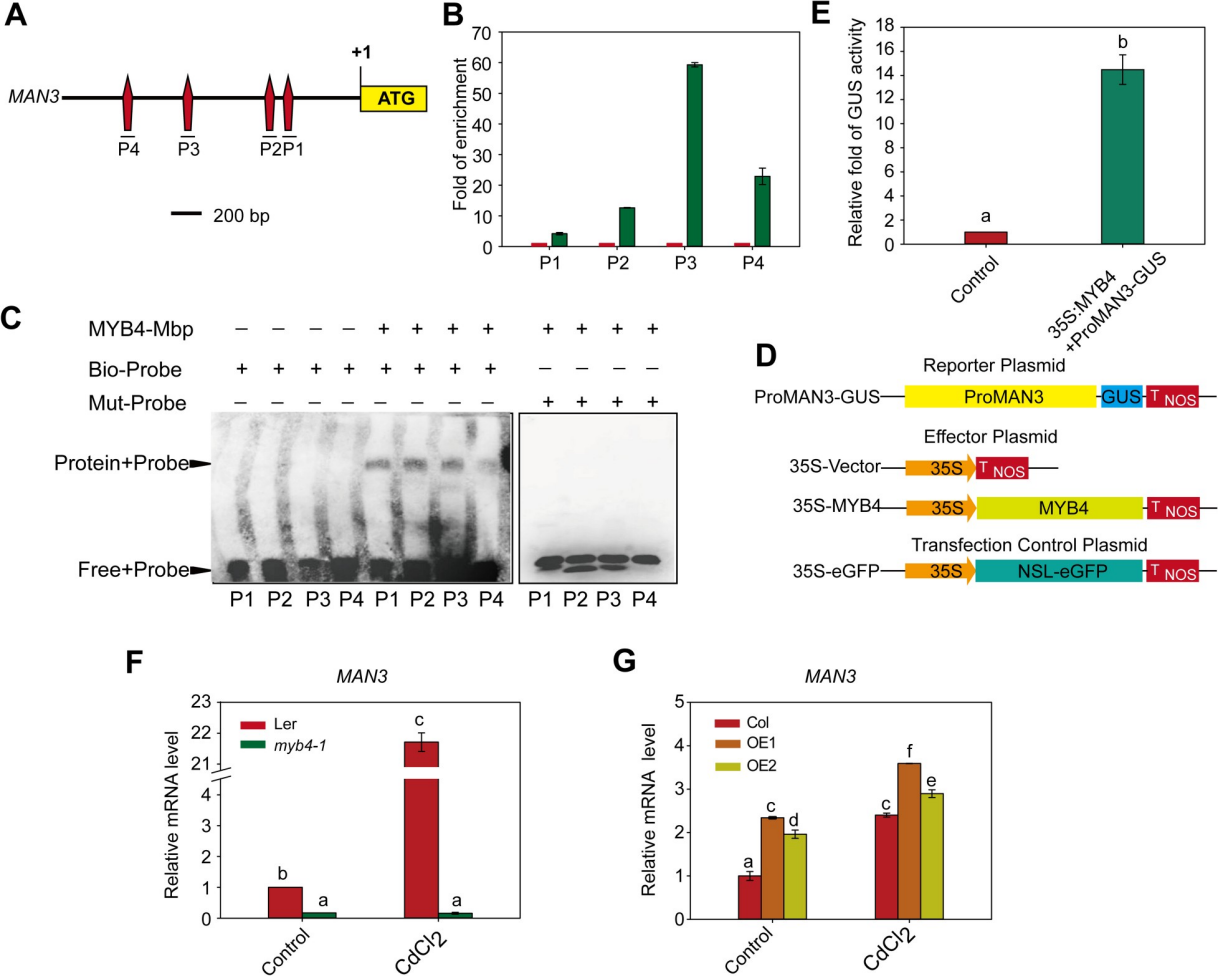
MYB4 directly binds to the promoters of *MAN3* gene and regulates its transcription. (A) Schematic diagram of the *MAN3* promoter. (B) qPCR analysis of DNA after ChIP experiments. Data are presented as means ±SD of three biological replicates. (C) EMSA analysis of the binding of recombinant MYB4 protein to the promoter of *MAN3* (P1-P4). The fragment in ProMAN3 (P1-P4) was used as Bio-probe. Labeled oligonucleotide (mut-P1, mut-P2, mut-P3 and mut-P4) was used as a mutant probe. (D) A schematic of the MAN3-promoter reporter construct, the effector plasmid, and the transfection control plasmid. *ProMAN3-GUS* plasmid represents GUS reporter driven by the full-length *MAN3* promoter. (E) Relative GUS activity of *ProMAN3-GUS* reporter after co-expression of *35S*:*MYB4* in *Nicotiana benthamiana*. 35S-empty vector was used as the effector plasmid control. Data are presented as means ± SD of three biological replicates. (F, G) qRT-PCR analysis of the *MAN3* transcript in the *myb4-1* mutant (E) and *MYB4-OE* lines (F). Ler, the *myb4-1* mutant, Col, and *MYB4-OE* lines were grown on 1/2 MS medium for 2 weeks, and treated with 50 μM CdCl_2_ for 12 hours and then their mRNAs were isolated for qRT-PCR analysis. *GAPDH* was used as an internal control. Data are presented as means ± SD of three biological replicates. Bars with different lowercase letters are significantly different at *P* < 0.05 (Tukey’s test).

The fact that the *MAN3* transcript is induced by Cd stress [37] promoted us to find the transcription factor upstream of *MAN3*. By searching promoter sequence, we identified four MYB4-binding sites in the promoter region of *MAN3* (Fig 4A). It was shown that MYB4 is a member of the MYB transcription factor family and localized in the nucleus [29]. The MYB4 gene contains two exons and one intron, and encodes a 282–amino acid protein located in cell nucleus (http://suba.live/suba-app/factsheet.html?id=AT4G38620). BLAST analysis of *MYB4* in the Nr (non-redundant) protein database of NCBI revealed that it shares >91% sequence similarity with other proteins such as a predicted transcription repressor MYB4 (*Camelina sativa*) protein (accession no.XP_010436960.1), a hypothetical (*Arabis alpina*) protein (AALP_AA7G264300; accession no.KFK30463.1), a predicted transcription repressor MYB4 (Raphanus sativus) protein (accession no.XP_018480221.1) and a predicted transcription repressor MYB4 (*Arabidopsis lyrata subsp*. *lyrata*) protein (accession no.XP_020872408.1; S10A and S10B Fig). The transcript of *MYB4* was detected in all tissues examined, with higher levels in silique and root (S10C Fig).

To determine whether MYB4 directly binds to the *MAN3* promoter, we performed chromatin immunoprecipitation (ChIP) assay, and found that MYB4 was able to bind to the P1-P4 regions in the promoter of *MAN3* (Fig 4B), which is further confirmed by EMSA assay (Fig 4C). To further determine whether the transcript of *MAN3* is regulated by MYB4, we created the *MAN3* promoter-driven *GUS* reporter gene (Fig 4D), and transiently co-expressed with the *MYB4* gene in *Nicotiana benthamiana* leaves and then determined the GUS activity. Co-expression of *ProMAN3-GUS* with *35S*:*MYB4* resulted in activation of the GUS reporter (Fig 4E), suggesting that MYB4 is a transcriptional activator of *MAN3*.

**Fig 5:**
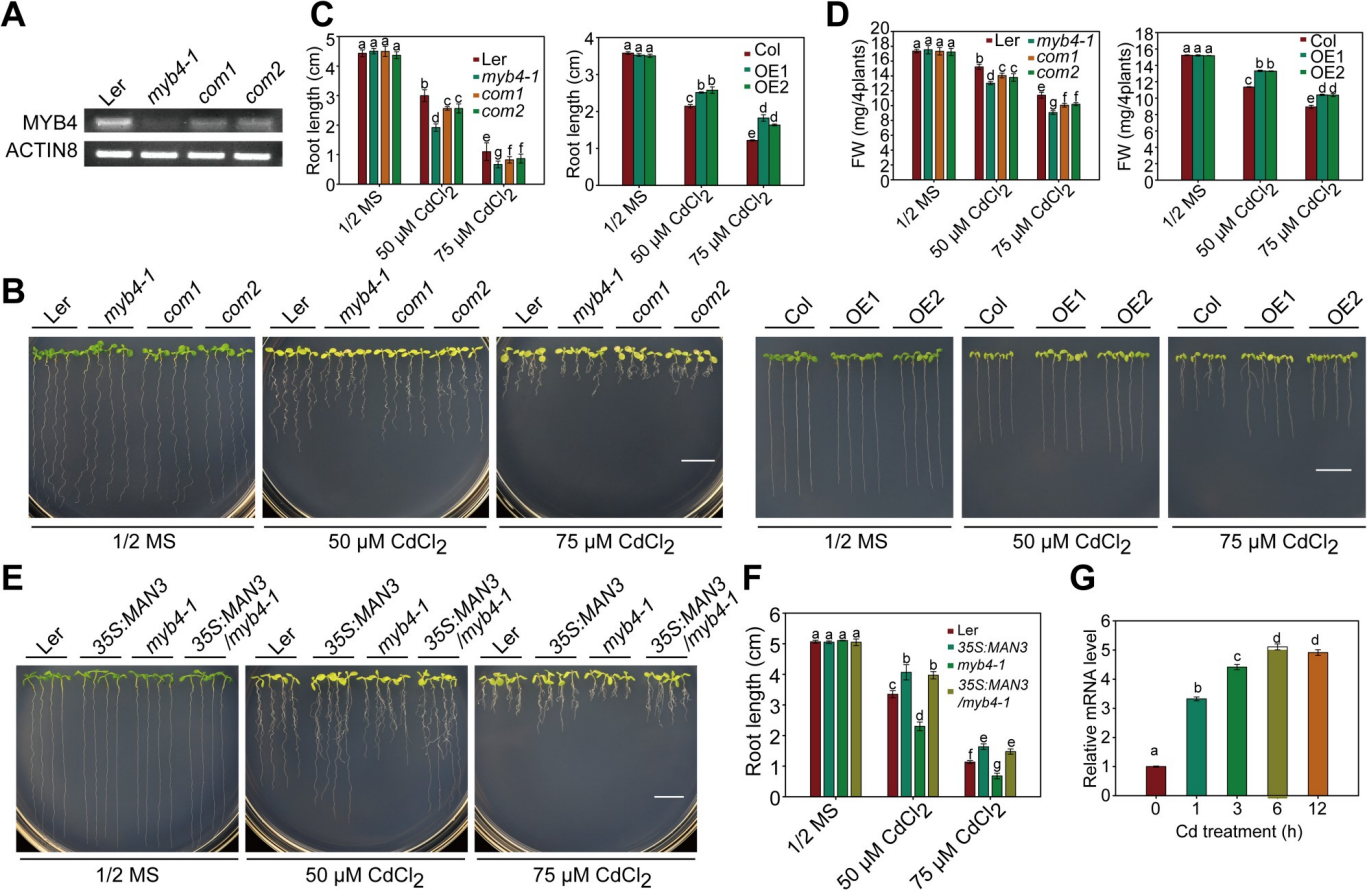
*MYB4* regulates Cd tolerance by activating *MAN3*. (A) RT-PCR analysis of the transcript level of *MYB4* in Ler, *myb4-1* and *myb4-1-*COM lines. *ACTIN8* was used as an internal control. (B) Cd tolerance of Ler, *myb4-1*, *myb4-1-*COM, Col, *MYB4-OE* lines seedlings. (C, D) Root length (C) and fresh weight (D) of plants described in (B). (E) Cd stress phenotypes of the wild-type Ler, *myb4-1*, *MAN3-OE*, *myb4-1/ MAN3-OE* plants. (F) Root length of plants described in (E). In B and E, three-day-old seedlings grown on 1/2 MS medium were transferred to 1/2 MS medium with or without 50 or 75 μM CdCl_2_ for about 2 weeks. Scale bar = 1 cm. In C, D and F, three independent experiments were done with similar results, each with three biological replicates. Four plants per genotype from one plate were measured for each replicate. Data are presented as means ± SD, n  =  3. Bars with different lowercase letters are significantly different at *P* < 0.05 (Tukey’s test). (G) Induction of *MYB4* by Cd stress. Wild-type plants were grown on 1/2 MS mediun for 2 weeks and then treated with 50 μM CdCl_2_ for 0, 1, 3, 6, 12 h before the tissues were harvested for RT-qPCR analysis. *GAPDH* was used as an internal control. Data are presented as means ± SD of three biological replicates.

To further determine whether MYB4 can activate the transcript of *MAN3 in planta*, we performed qRT-PCR analysis using Ler, *myb4-1* (Fig 5A), Col, and *35S*:*MYB4* plants (S12 Fig). The data showed that in the absence or presence of Cd stress, the transcript of *MAN3* was dramatically downregulated in the *myb4-1* mutant (Fig 4F), but was significantly upregulated in *MYB4-OE* plants (Fig 4G). Collectively, these data provide evidence that MYB4 regulates the *MAN3* transcription by directly binding to its promoter.

### MYB4 acts as upstream of MAN3 in the GSH-depenent PC synthesis pathway

Based on the fact that MYB4 directly regulates expression of *MAN3*, we next determined whether altered expression of *MYB4* affects plant Cd tolerance. We identified the *myb4-1* mutant (Alonso et al., 2003; CS26404, Ler background; Fig 5A), and analyzed its Cd tolerance (Fig 5B). The *myb4-1* mutant showed similar phenotypes to the growth and development of the Ler (S2B Fig). However, Cd tolerance was markedly reduced in *myb4-1* mutant plants compared with wild-type plants (Fig 5B), as indicated by root length and fresh weight (Fig 5C and 5D). We further generated genetically complemented *myb4-1* mutant plants, and found that the complemented lines (COM1 and COM2; Fig 5A) reversed the Cd-sensitive phenotype of the *myb4-1* mutant to the level of the Ler (Fig 5B–5D), indicating that loss-of-function of *MYB4* is responsible for the Cd-sensitive phenotype. We also analyzed the responses of the *myb4* mutant to other stresses, including Na_3_AsO_4_, ZnSO_4_, CuSO_4_, and Pb(NO_3_)_2_, and observed that *myb4-1* mutant showed enhanced sensitive to Pb but not to As, Zn, and Cu (S11 Fig).

To further determine the role of *MYB4* in plant Cd tolerance, we generated more than nine *MYB4*-overexpression lines, and chose two lines OE1 and OE2 to test their tolerance (S12 Fig). Enhanced Cd tolerance was observed in *MYB4-OE* plants compared with wild-type plants (Fig 5B), as demonstrated by root length and fresh weight (Fig 5C and 5D). Taken together, all these results suggest that *MYB4* is a positive regulator of plant Cd tolerance.

To investigate the genetic epistasis between *MYB4* and *MAN3*, we introduced *35S*:*MAN3* construct into the wild type Ler and the *myb4-1* mutant, and the resulting *35S*:*MAN3/myb4-1* plants (S5C and S5D Fig) showed similar Cd-tolerant phenotype as *35S*:*MAN3* plants (Fig 5E), as indicated by root length (Fig 5F). Moreover, we found that Cd-induced transcript of *MYB4* is earlier than that of *MAN3* (Fig 5G) [37]. These results suggest that *MAN3* is epistatic to *MYB4*.

**Fig 6:**
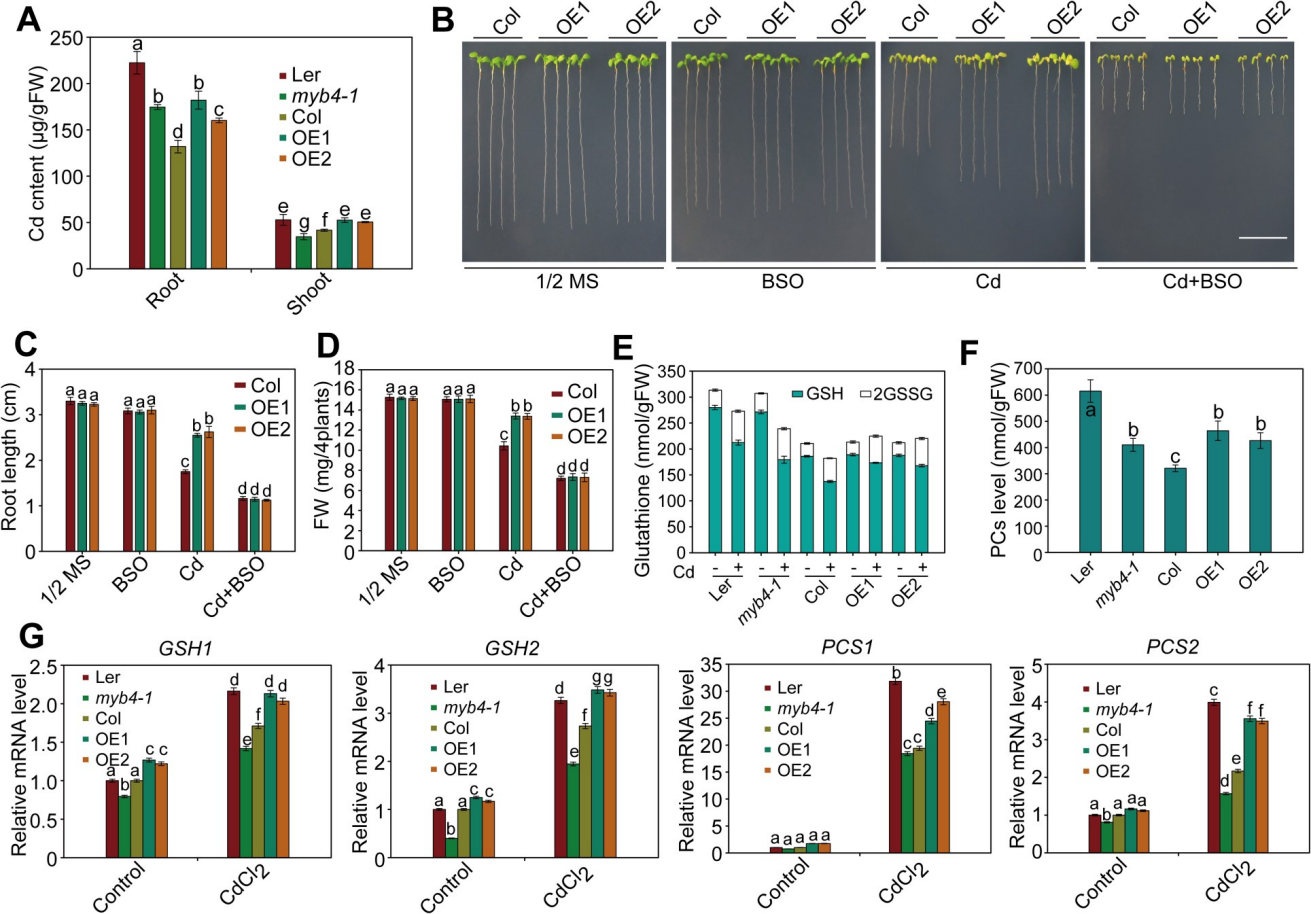
*MYB4* positively regulates Cd tolerance by the GSH-dependent pathway. (A) Analysis of Cd contents of Ler, *myb4-1*, Col, *MYB4-OE* lines. Plants were grown on 1/2 MS medium with or without 50 μM CdCl_2_ for 2 weeks, and their roots and shoots were sampled for measurement of Cd content, respectively. Data are presented as means ± SD of three biological replicates. Bars with different lowercase letters are significantly different at *P* < 0.05 (Tukey’s test). (B) Effects of BSO in Col and *MYB4-OE* lines under Cd stress. Three-day-old seedlings grown on 1/2 MS medium were transferred to 1/2 MS medium with or without 50 μM CdCl_2_ and 0.1 mM BSO for about 2 weeks. (C, D) Root length and fresh weight of plants described in (B). Three independent experiments were done with similar results, each with three biological replicates. Four plants per genotype from one plate were measured for each replicate. Data are presented as means ± SD, n  =  3. Bars with different lowercase letters are significantly different at *P* < 0.05 (Tukey’s test). (E, F) Total glutathione content and total PC content in Ler, *myb4-1*, Col, *MYB4-OE* lines. Two-week-old plants grown on 1/2 MS medium were treated with or without 50 μM CdCl_2_ for 24 h for analysis of glutathione and PC contents. Data are presented as means ± SD, n  =  3. Bars with different lowercase letters are significantly different at *P* < 0.05 (Tukey’s test). (G) Transcript levels of genes involved in the PC synthesis pathway in Ler, *myb4-1*, Col, *MYB4-OE* plants in the absence or presence of Cd. Two-week-old plants grown on 1/2 MS medium were treated with or without 50 μM CdCl_2_ for 6 h for analysis of transcript levels of genes. *GAPDH* was used as an internal control. Data are presented as means ± SD, n  =  3.

To further determine whether *MYB4* regulates Cd tolerance through *MAN3*-mediated GSH-dependent pathway, we first measured Cd contents of Ler, *myb4-1*, Col, *MYB4-OE* seedlings under Cd tress, and found that Cd contents were significantly lower in root and shoot of *myb4-1* plants, but was markedly higher in those of *MYB4-OE* plants compared with wild-type (Fig 6A). We then used BSO to treat wild-type and *MYB4*-overexpressing plants. This result showed that the Cd-tolerant phenotype in *MYB4*-overexpressing plants disappeared when BSO was added together with CdCl_2_ (Fig 6B), as determined by root length (Fig 6C) and fresh weigh (Fig 6D).

Analysis of GSH and PCs levels further showed that significant differences were observed in GSH (Fig 6E) and PCs (Fig 6F) levels between Ler and *myb4-1*, Col-0 and *MYB4-OE* seedlings under Cd tress. Finally, we analyzed the transcript levels of genes *GSH1*, *GSH2*, *PCS1*, and *PCS2* in Ler, *myb4-1*, Col and *MYB4-OE* seedlings in response to Cd stress. The transcript levels of *GSH1*, *GSH2*, *PCS1*, and *PCS2* were significantly lower in the *myb4-1* mutants, but were markedly higher in the *MYB4-OE* lines than in the wild type (Ler and Col, respectively) in the absence or presence of Cd stress (Fig 6G). In addition, we also analyzed the transcript levels of other Cd stress-related genes, including *GR1*, *GR2*, *ABCC1*, *ABCC2* and *PDR8*. The transcript levels of *GR1* and *PDR8* were consistent with differences of *GSH1*, *GSH2*, *PCS1*, and *PCS2* but the transcript levels of *GR2*, *ABCC1* and *ABCC2* showed no regular difference in the absence or presence of Cd stress (S13 Fig). Taken together, all these results suggest that *MYB4* positively regulates Cd tolerance by the *MAN3*-mediated GSH-dependent PC synthesis pathway.

### MAN3-Mannose-MNB1 is involved in regulation of MYB4-mediated Cd tolerance

**Fig 7:**
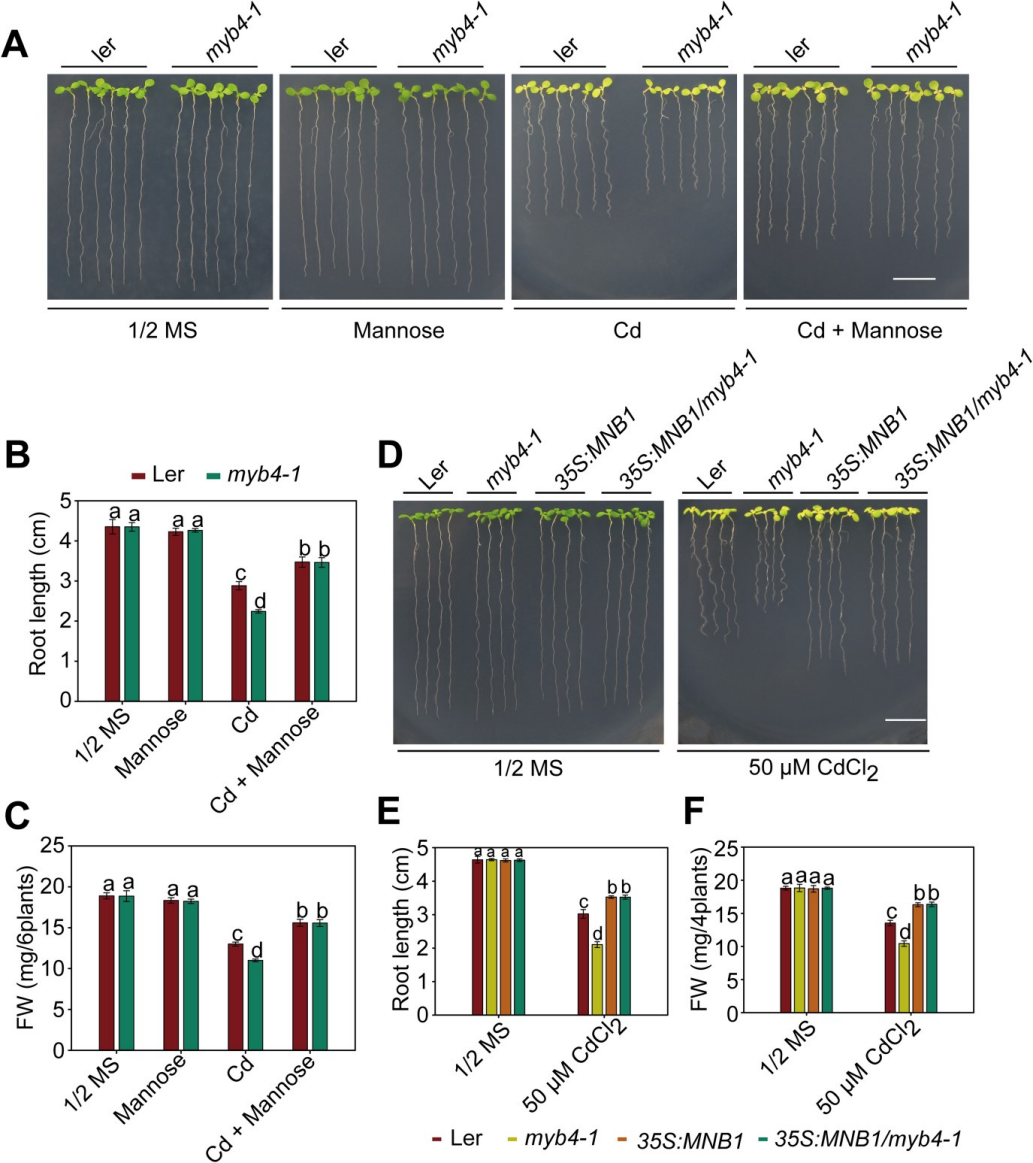
MAN3-Mannose-MNB1 is involved in regulation of MYB4-mediated Cd tolerance. (A) Analysis of Cd tolerance in Ler and *myb4-1* mutant seedlings in the absence or presence of Cd and mannose. (B, C) Root length and fresh weight of plants described in (A). (D) Cd stress phenotypes of the Ler, *myb4-1*, *MNB1-OE*, and *MNB1-OE/myb4-1* seedlings. (E, F) Root length and fresh weight of plants described in (D). In (A) and (D), three-day-old seedlings grown on 1/2 MS medium were transferred to 1/2 MS media with or without 50 μM CdCl_2_ or 1.5 mM mannose for about 2 weeks. Scale bar = 1 cm. In (B), (C), (E), and (F), three independent experiments were done with similar results, each with three biological replicates. Four plants per genotype from one plate were measured for each replicate. Data are presented as means ± SD, n  =  3. Bars with different lowercase letters are significantly different at *P* < 0.05 (Tukey’s test).

Based on the fact that *MYB4* acts upstream of *MAN3* to positively regulate Cd tolerance through the GSH-dependent PC synthesis pathway, we proposed that MAN3-mediated mannose may function as downstream of MYB4 to modulate Cd tolerance. To test this possibility, we used exogenous mannose to treat the wild-type Ler and the *myb4-1* mutant, and found that, when exogenous mannose was added together with CdCl_2_, reduced Cd-tolerance in *myb4-1* plants restores to the level of the wild-type Cd-tolerance (Fig 7A), as determined by root length (Fig 7B) and fresh weigh (Fig 7C). To determine whether galactose and glucose have similar protective effects of wild-type and *myb4-1* mutant against Cd toxicity, we also tested the effects of galactose and glucose in wild-type and *myb4-1* mutant plants to Cd toxicity. We found that these compounds did not confer protective effects of wild-type and *myb4-1* mutant plants against Cd toxicity under our experimental conditions (S14A and S14B Fig). These results suggest that mannose is involved in MYB4-mediated Cd tolerance.

To further determine whether *MNB1* acts downstream of *MYB4* to positively regulate Cd tolerance, we introduced *35S*:*MNB1* construct into the wild type Ler and the *myb4-1* mutant (S5D and S5E Fig), and the resulting *35S*:*MNB1/myb4-1* plants showed similar Cd-tolerance to *35S*:*MNB1* plants (Fig 7D), as indicated by root length (Fig 7E) and fresh weight (Fig 7F), suggesting that *MNB1* is epistatic to *MYB4*. In addition, we also constructed the double mutant *35S*:*MYB4/mnb1-1* and analyzed its cadmium stress phenotype. As shown in S14C and S14D Fig, the resulting *35S*:*MYB4/mnb1-1* plants showed similar Cd- sensitivity to *mnb1-1* plants, suggesting that MYB4-mediated Cd tolerance is dependent on MNB1. Taken together, all these results suggest that MYB4 regulates Cd tolerance through a MAN3-Mannose-MNB1 regulation module.

## Discussion

Plants have various strategies in respond to Cd stress for detoxification and tolerance [7,41,42]. The GSH-dependent PCs synthesis pathway is one of the most important mechanisms in Cd detoxification and tolerance in plants [7,41]. In the previous studies, we found that MAN3-mediated mannose triggers GSH-dependent PCs synthesis pathway contributing to the Cd accumulation and detoxification [37]. In this study, we have elucidated the detailed mechanisms of MAN3-mediated Cd tolerance through mannose-binding to the GNA-related domain of MNB1, a homologous mannose-binding lectin in Arabidopsis of CaMBL1 [32], to trigger the GSH-dependent PC synthesis pathway. Moreover, we also demonstrated that, in response to Cd stress, MYB4 acts upstream of MAN3 to positively regulate the Cd accumulation and tolerance. Our results showed that an *Arabidopsis* MYB4-MAN3-Mannose-MNB1 signaling cascade is involved in the regulation of plant Cd tolerance.

Mannose-binding lectins play an important role in plant defense signaling during pathogen attack [32]. It was found that the pepper mannose-binding lectin CaMBL1 is required to regulate cell death and defense responses to microbial pathogens [32], and mannose has been shown to bind to plant lectins that possess diverse biological roles [34–36]. So far, to our knowledge, no plant lectin has been identified to modulate heavy metal detoxification. In this study, we demonstrated that the *Arabidopsis* mannose-binding lectin MNB1 positively regulates the Cd accumulation and tolerance through the GSH-dependent Cd-activated PC synthesis pathway. First, the expression level of *MNB1* was significantly induced by Cd stress, and loss-of-function of *MNB1* displayed decreased Cd accumulation and tolerance, whereas overexpression of *MNB1* enhanced Cd accumulation and tolerance. Second, we found that mannose is able to bind to the GNA-related domain of MNB1, which is a conserved domain in plant mannose-binding lectins [33,34]. MNB1 deletion mutants that contain full GNA-related lectin domain could bind to D-mannose, whereas MNB1 deletion mutants without GNA-related lectin domain are not able to bind to D-mannose, suggesting that the GNA-related domain of MNB1 is essential for its binding to D-mannose. Because it was shown that MAN3-mediated mannose plays an important role in plant responses to Cd stress [37], we further demonstrated that *MNB1* is involved in regulating MAN3-mediated Cd tolerance, and mannose binding to the GNA-related domain of MNB1 is required for MAN3-mediated Cd tolerance. Finally, we analyzed Cd contents in WT, *mnb1* mutants and *MNB1-OE* lines under Cd stress, and found Cd content was significantly lower in the *mnb1* mutants but markedly higher in the *MNB1-OE* lines than WT under Cd stress, suggesting MNB1-mediated Cd tolerance is involved in a mechanism of Cd accumulation and tolerance. In addition, we also found that MNB1-mediated Cd tolerance phenotype could be eliminated in the presence of BSO, a GSH synthesis inhibitor. The expression of the genes involved in the GSH-dependent PCs synthesis pathway was significantly lower in the *mnb1* mutants but markedly higher in the *MNB1-OE* lines than WT under Cd stress, which was consistent with the differences of GSH content and PC content, suggesting a GSH-dependent PCs synthesis pathway is indeed involved in the mechanism of MNB1-meidated Cd tolerance. Recently, it was found that the potential role of SLIM1 in Cd tolerance of plants by inducing -S responses in the cell caused by depleting the GSH pool [52], and that, in addition to glutathione depletion, an enhanced oxidative state and depletion of upstream thiols, are necessary to induce the transcription of sulfate assimilation genes during early cadmium stress [53]. Therefore, it is of interest to further study to determine whether MNB1 affects sulfate assimilation, thereby altering reactive oxygen species signaling and regulating gene expression of GSH-dependent PCs synthesis pathway. In addition, in this study, we also analyzed whether *MNB1* is involved in regulating other stress responses, such as Cu, Pb, As and Zn, and found that *mnb1* mutants showed enhanced sensitive to Cu and Pb but not to As and Zn, suggesting that *MNB1* may also be involved in the regulation of Cu and Pb stress responses. Therefore, it will be interesting to investigate the mechanisms of MNB1-mediated Cu and Pb tolerance.

Many transcription factors have been showed to be induced by heavy metal stress in plants [23,54]; however, only a few transcriptional factors were identified as the regulators of Cd detoxification and tolerance in plants, including HsfA4a [23], bHLH29, bHLH38, and bHLH39 [24], ZAT6 [25], OXIDATIVE STRESS2 [26], WRKY13 [55], WRKY12 [56], and MYB49 [29]. MYB4 is a member of MYB transcription factor family involved in the regulation of secondary metabolism and cell shape, disease resistance and responds to different stresses [30]. In this study, we demonstrated that MYB4 transcription factor directly binds to the promoter of *MAN3* to positively regulate the transcript of *MAN3* by several biochemical experiments. Although we failed in yeast one-hybrid, ChIP-qPCR assay showed that MYB4 can specifically bind to the promoter of *MAN3* gene, and transient expression experiments and qRT-PCR analysis also showed that MYB4 positively regulates the transcript of *MAN3*. Moreover, MYB4 positively regulates Cd accumulation and tolerance through the GSH-dependent Cd-activated PC synthesis pathway, and genetics analysis showed that MYB4 act upstream of MAN3 and MNB1 to positively regulates Cd accumulation and tolerance. In addition, we also tested the responses of the *myb4-1* mutant to As, Zn, Cu, and Pb stresses. Interestingly, we found that, unlike *mnb1* mutants, *myb4-1* mutant showed enhanced sensitive to Pb but not to As, Zn, and Cu, suggesting that *MYB4* and *MNB1* have cross and different functions in regulating heavy metal stress responses.

**Fig 8:**
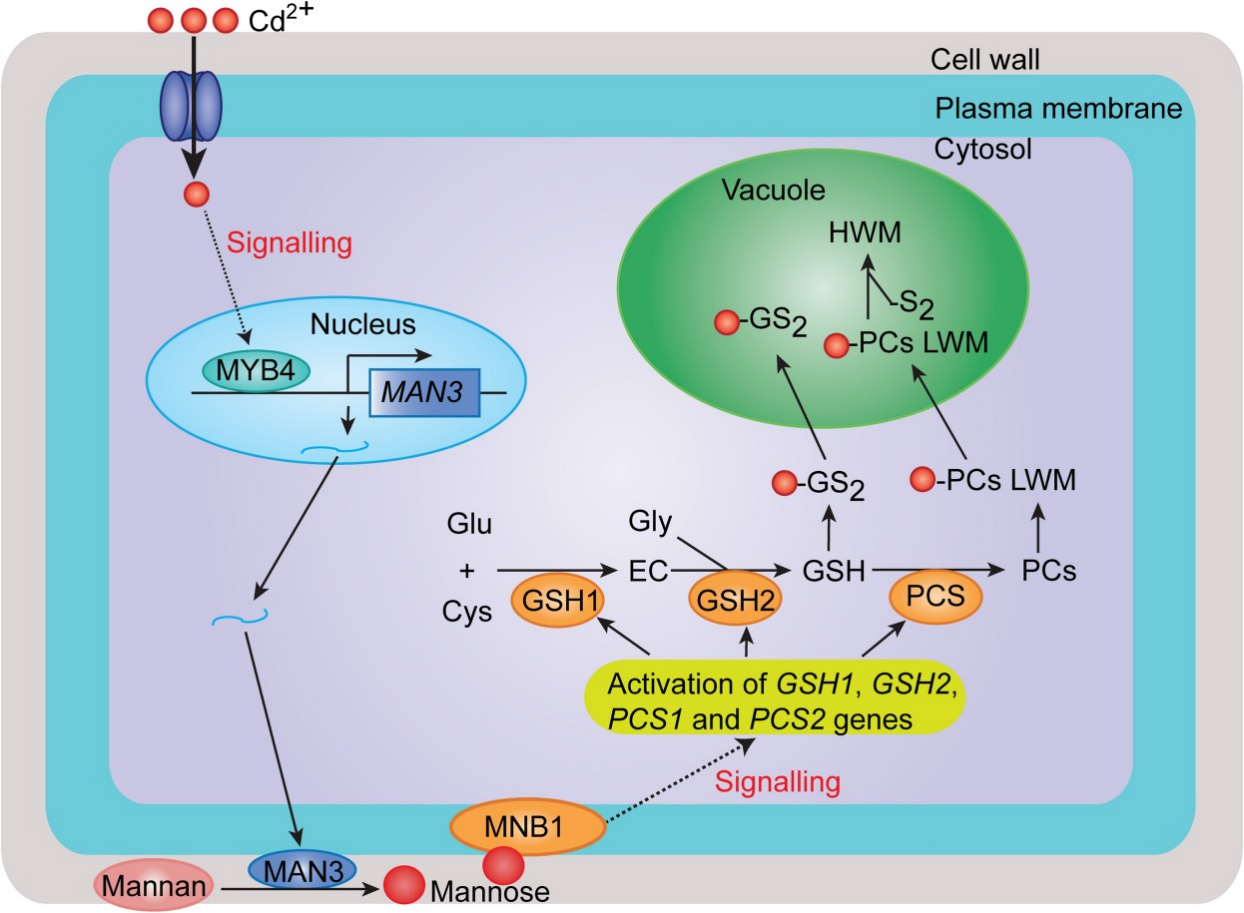
A proposed model of MYB4-mediated MAN3-Mannose-MNB1 Signaling Cascade for regulation of Cd tolerance in *Arabidopsis*. In response to Cd stress, MYB4 is rapidly induced, and directly activates the expression of *MAN3* and then enhances mannose binding to MNB1, which in turn triggers GSH-dependent Cd-activated PC synthesis by coordinated activation of genes expression, and thereby leads to increased Cd accumulation and tolerance.

In summary, we provide compelling evidence supporting the fact that MYB4-MAN3-Mannose-MNB1 signaling cascade regulates cadmium tolerance in *Arabidopsis* through the GSH-dependent PC synthesis pathway. Cd stress quickly induces the expression of *MYB4*, which directly activates the expression of *MAN3* to increase mannose content, and thus enhances mannose binding to MNB1, which in turn triggers GSH-dependent Cd-activated PC synthesis by coordinated activation of genes expression, and thereby leads to increased Cd accumulation and tolerance (Fig 8).

## Materials and methods

### Plant materials and growth conditions

The T-DNA insertion lines for *mnb1* mutants (*mnb1-1*:SALK-038821C; *mnb1-2*: SALK-121641; ecotypes Columbia Col-0) [38], and Transposable Elements Insertion *myb4-1* mutant(CS26404, Landsberg erecta [Ler-0]) were obtained from the Arabidopsis Biological Resource Centre (ABRC) at Ohio State University, USA.

The Arabidopsis seeds were surface-sterilized and planted on half-strength Murashige-Skoog (1/2 MS) media [57] containing 1% sucrose and 1.2% agar (Sangon Biotech) (pH 5.7). They were vernalized in the dark at 4°C for 3 d, and then grew in a controlled environment with 16 h light/8 h dark cycles at 22°C. Three-day-old wild-type and mutant or transgenic seedlings were transferred to 1/2 MS agar plates in the absence or presence of heavy metal or other supplements for the indicated number of days.

### Vector construction and plant transformation

The MAN3:GUS construct was generated as described by Chen et al. [25]. To generate MNB1-overexpressing (MNB1-OE), MYB4-overexpressing (MYB4-OE), MAN3-overexpressing (MAN3-OE), and MNB1-green fluorescent protein (GFP) transgenic plants, cDNA of full-length MNB1, MYB4 or MAN3 was amplified with specific primers by RT-qPCR (primers are listed in S1 Table), and these products were cloned into the pCAMBIA1301 or pXB94 (pART27 with expanded restriction sites, 35S promoter, and GFP reporter) at the XbaI, Kpn1 and Xho1 restriction enzymes (named 35S::MYB4 and 35S::MNB1::GFP), and transformed into Arabidopsis plants by using the floral dip method [58]. All the obtained transgenic lines used in this study were selected T3 homozygous plants with single copy insertion.

### Subcellular localization of 35S:MNB1-GFP

The transient expression experiments of MNB1-GFP were done in *Nicotiana benthamiana*, as previously described [59]. The results of subcellular localization were observed using confocal microscopy (Leica TCS-SP8; Wetzlar, Hesse-Darmstadt, Germany).

### SEM analysis

Two-week-old plants grown on1/2 MS medium, samples (Col and *mnb1* mutant seedlings) were fixed in 2.5% glutaric dialdehyde. The cell wall structure observation of Col and *mnb1* mutant by a tungsten filament scanning electron microscope (JSM-6490LV, SEM, Japan), as previously described [60,61]. Micrographs of each sample were observed at× 200 and × 300 magnifications.

### Semi-quantitative RT-PCR and quantitative real-time RT-PCR

Plant total RNA was extracted from 2-week-old seedlings using Trizol Reagent (Invitrogen, CA, USA), and the first-stand cDNA was synthesized by total RNA with SuperScript II RNase H2 reverse transcriptase (Invitrogen) using random hexamer primer (Promega). Semi-quantitative RT-PCR was carried out using gene-specific primers. *ACTIN8* was used as an internal control. RT-qPCR was performed according to the instructions provided for the Bio-Rad iCycler iQ system (Bio-Rad Laboratories, CA, USA) using platinum SYBR Green qPCR SuperMix-UDG (Invitrogen). The fold change of transcripts was calculated based on an efficiency calibrated model [62] and each sample were quantified at least in triplicate and normalized using glyceraldehyde 3-phosphate dehydrogenase (GAPDH) gene as an internal control. Statistical differences between the samples were evaluated by Student’s t-test or ANOVA in combination with post-hoc test using delta Ct values [62]. The specific primers used are listed in S1 Table.

### Measurement of Cd and GSH/GSSG/PC content

Seeds were grown on 1/2 MS medium with or without 50 μM CdCl_2_ for two weeks and their seedlings were then sampled for analysis of Cd content. Cd content was carried out according to the method described by Lee et al. [14] (2003). Digested samples were measured using an atomic absorption spectrometer (Solaar M6;Thermo Fisher). Two-week-old seedlings grown on 1/2 MS medium were treated with or without 50 μM CdCl_2_ for 24 h and then sampled for analysis of GSH/GSSG/PC contents. GSH/GSSG/PC contents were quantitated as described by Chen et al. [37].

### Transient expression assays in *Nicotiana benthamiana*

Transient expression assays were carried out as described in previous studies [25,59,63] (Sparkes et al., 2006; Chen et al., 2009; Chen et al., 2016). A. tumefaciens cells were obtained by centrifugation and suspended in the solutions containing 50 mM MES, 5 g/L D-Glc, 2 mM Na_3_PO_4_, and 0.1 mM acetosyringone to an optical density (600 nm) value of 0.1. *A*. *tumefaciens* cells were incubated at room temperature for 4 h and then co-transformed into epidermal cells of *Nicotiana benthamiana* using a needle-free syringe. The GUS staining and GUS activity measurements were analyzed at 48 h after injection, according to published protocols [64].

### ChIP-qPCR assay

ChIP assay was performed according to the protocol described previously with slight modifications [65]. To determine whether GFP fusion affects the function of MYB4, we analyzed the phenotypes of *35S*: *MYB4* and 35S:MYB4-GFP plants under Cd stress, and found that both of them were more tolerant to Cd than wild-type plants, and there was no significant difference between them (S15 Fig), indicating that GFP fusion did not affect the function of MYB4. Therefore, we selected 35S:MYB4-GFP plants for CHIP analysis. Briefly, about 3 g of 15-day-old 35S::MYB4:GFP transgenic plants as the experimental group and 35S::GFP plants as the control group were cross-linked by formaldehyde (3%) for 30 min, which was stopped by the addition of glycine to a final concentration of 50mM for 2 min. The purified cross-linked nuclei were sonicated to shear the chromatin into 0.2–1.2 kb fragments, which were divided into three parts, one part was used for input DNA, and the two other parts were incubated with anti-GFP antibody (Abmart, China).The antibody-bound complex was precipitated with Protein A agarose beads. The DNA fragments in the immunoprecipitated complexes were released by reversing the cross-linking at 65°C for 6 h. Purified immunoprecipitated DNA was analyzed by qPCR using primers listed in Supplementary S1 Table.

### Electrophoresis mobility shift Assay (EMSA)

EMSA was conducted using a LightShift Chemiluminescent EMSA Kit (Thermo Fisher Scientific, https://www.thermofisher.com/) following the manufacturer’s protocol. The recombinant protein *MYB4-MBP* was expressed and purified from *Escherichia* coli BL21 (DE3) cell line. Oligonucleotide probes (ProMAN3 P1 and P4) were synthesized and labelled with biotin at the 3′hydroxyl end of the sense strand. All oligonucleotide sequences used here are listed in S1 Table.

### Yeast one-hybrid assay

The CDS of MYB4 was ligated into the PJG4-5 vector. The fragment of the MAN3 promoter was cloned into the placZi vector. All primers used are listed in S1 Table. The Y1H assay was conducted as previously described [66].

### Expression of the MNB1 Protein, immunoblot analysis and its binding assay with man-agarose

The MNB1 gene was cloned into the vector pET22b to generate pET22b::MNB1, which produced a translational fusion of MNB1 to a His tag at the N terminus. The MNB1 protein construct was expressed in Escherichia coli and purified on a Man-agarose column. The MNB1 deletion series was made by PCR amplification using the primers showed in S1 Table. To express the MNB1 gene, pET22b::MNB1 was transformed into E. coli Rosetta (DE3).Recombinant strains were grown in 200 mL of Luria-Bertani medium at 37°C to an optical density (600 nm) value of 0.5,induced with 1mM isopropyl-D-thiogalactopyranoside for 12h at 16°C,and harvested. Cells were collected by centrifugation, resuspended in 20 mL of PBS buffer (137 mM NaCl, pH 7.4, 2.7 mM KCl,10 mM Na_2_HPO_4_,2mM KH_2_PO_4_), and disrupted by sonication. Cell debris was removed by centrifugation, and the supernatant was used to a D-Man-agarose column with purification (Sigma) according to the instructions of the manufacturer. Purified MNB1 proteins (10–50 mg) were separated by 12% SDS-PAGE and transferred to nitrocellulose membranes (Sangon Biotech) by electro-blotting. For detection of proteins, an anti-His antibody (Solarbio) was used at 1:3,000 dilution.

## Supporting information

S1 Fig Expression patterns of *MNB1* gene. (A) Phylogenic tree of *MNB1*. (B) Similarity in protein sequences between MNB1 and other proteins. (C) RT-qPCR analysis of *MNB1* transcript level in different tissues of wild-type plants. RNA was isolated from roots, rosette leaves, cauline leaves, inflorescence, stem, siliques of the wild-type plants. *GAPDH* was used as an internal control. Data are presented as means ± SD of three biological replicates. (D) Schematic of T-DNA insertion sites on the locus of *MNB1* gene in the *mnb1* mutants. (E) Subcellular localization of MNB1. (F) Observation of the cell wall structure of Col and *mnb1* mutant by SEM.
(TIF)

S2 Fig Growth of *mnb1* and *myb4* mutants. (A) Growth of 6-week-old /4-week-old Col and the *mnb1* mutants. (B) Growth of 6-week-old /4-week-old Ler and the *myb4-1* mutant. Scale bar = 1 cm.
(TIF)

S3 Fig Tolerance of Col and *mnb1* mutant lines to As, Pb, Zn or Cu stress. Three-day-old seedlings grown on 1/2 MS medium were transferred to 1/2 MS medium with or without 100 μM Na3AsO4, 600 μM Pb(NO3)2, 200 μM ZnSO4 or 100 μM CuSO4 for about 2 weeks. Scale bar = 1 cm.
(TIF)

S4 Fig Growth of *MNB1*-overexpression lines. (A) Growth of 6-week-old /4-week-old Col or *MNB1*-overexpression lines. Scale bar = 1 cm. (B) qRT-PCR analysis of the transcript level of *MNB1* in Col and *MNB1*-overexpression lines. *GAPDH* was used as an internal control. Data are presented as means ± SD of three biological replicates.
(TIF)

S5 Fig Transcript levels of other genes involved in the PC synthesis pathway in Col, *mnb1* mutants and *MNB1-OE* plants. Two-week-old plants grown on 1/2 MS medium were treated with or without 50 μM CdCl_2_ for 6 h for analysis of transcript levels of genes. *GAPDH* was used as an internal control. Data are presented as means ± SD, n  =  3.
(TIF)

S6 Fig Nucleotide and deduced amino acid sequences of AtMNB1 cDNA encoding a GNA-related lectin protein. The deduced amino acid sequences by red letters are below the nucleotide sequences by black letters. The signal peptide sequence is on the yellow background. The putative GNA-related lectin is underlined and bold type and the putative N-glycosylation sites are on the red background. The termination codon is marked by an asterisk (*) on a green background.
(TIF)

S7 Fig Identification of transgenic plants materials by RT-qPCR analysis of transcript levels of *MNB1*, *MYB4* and *MAN3*. *GAPDH* was used as an internal control. Data are presented as means ± SD of three biological replicates.
(TIF)

S8 Fig The GNA-related domain of MNB1 is required for Mannose-mediated Cd tolerance. (A) Effect of 1.5 mM mannose treatment on Cd tolerance of the Col, *mnb1*, *35SMNB1*:*mnb1-COM* and *MNB1*^Δ^/*mnb1* seedlings with or without 50 μM CdCl_2_. Three-day-old seedlings grown on 1/2 MS medium were transferred to 1/2 MS medium with or without 50 μM CdCl_2_ or 1.5 mM mannose for about 2 weeks. Scale bar = 1 cm. (B, C) Root length (B) and fresh weight (C) of plants described in (A). Three independent experiments were done with similar results, each with three biological replicates. Four plants per genotype from one plate were measured for each replicate. Data are presented as means ± SD, n  =  3. Bars with different lowercase letters are significantly different at *P* < 0.05 (Tukey’s test).
(TIF)

S9 Fig The GNA-related domain of MNB1 is required for MAN3-mediated Cd tolerance. (A) Cd tolerance of the Col, *mnb1*, *MAN3-OE*, *MAN3-OE/mnbl1*, *MAN3-OE/35SMNB1*:*mnb1-COM* and *MAN3-OE/MNB1*^*Δ*^*/mnb1* seedlings. Three-day-old seedlings grown on 1/2 MS medium were transferred to 1/2 MS medium with or without 50 μM CdCl_2_ for about 2 weeks. Scale bar = 1 cm. (B, C) Root length (B) and fresh weight (C) of plants described in (A). Three independent experiments were done with similar results, each with three biological replicates. Four plants per genotype from one plate were measured for each replicate. Data are presented as means ± SD, n  =  3. Bars with different lowercase letters are significantly different at *P* < 0.05 (Tukey’s test).
(TIF)

S10 Fig Expression patterns of *MYB4* gene. (A) Phylogenic tree of *MYB4*. (B) Similarity in protein sequences between MYB4 and other proteins. (C) RT-qPCR analysis of *MYB4* transcript level in different tissues of wild-type plants. RNA was isolated from roots, rosette leaves, cauline leaves, inflorescence, stem, siliques of the wild-type plants. *GAPDH* was used as an internal control. Data are presented as means ± SD of three biological replicates.
(TIF)

S11 Fig Tolerance of Ler and *myb4-1* lines to As, Cu, Zn or Pb stress. Three-day-old seedlings grown on 1/2 MS medium were transferred to 1/2 MS medium with or without 100 μM Na_3_AsO_4_, 600 μM Pb(NO_3_)_2_, 200 μM ZnSO_4_ or 100 μM CuSO_4_ for about 2 weeks. Scale bar = 1 cm.
(TIF)

S12 Fig Growth of *MYB4*-overexpression lines. (A) Growth of 6-week-old /4-week-old Col or *MYB4*-overexpression lines. Scale bar = 1 cm. (B) qRT-PCR analysis of the transcript level of *MYB4* in Col and *MYB4*-overexpression lines. *GAPDH* was used as an internal control. Data are presented as means ± SD of three biological replicates.
(TIF)

S13 Fig Transcript levels of other Cd stress-related genes in Ler, *myb4-1*, Col, *MYB4-OE* plants. Two-week-old plants grown on 1/2 MS medium were treated with or without 50 μM CdCl_2_ for 6 h for analysis of transcript levels of genes. *GAPDH* was used as an internal control. Data are presented as means ± SD, n  =  3.
(TIF)

S14 Fig MYB4 is involved in regulation of Mannose-mediated Cd tolerance. (A) Analysis of Cd tolerance in Ler and *myb4-1* mutant seedlings in the absence or presence of Cd, galactose, and glucose. (B) Root length of plants described in (A). (C) Cd and mannose stress phenotypes of the Col, *mnb1-1*, *MYB4-OE*, and *MYB4-OE*/*mnb1-1* seedlings. (D) Root length of plants described in (C). In (A) and (C), three-day-old seedlings grown on 1/2 MS medium were transferred to 1/2 MS medium with or without 50 μM CdCl_2_, 1.5 mM mannose, 1.5 mM galactose, or 1.5 mM glucose, for about 2 weeks. Scale bar = 1 cm. In (B) and (D), three independent experiments were done with similar results, each with three biological replicates. Four plants per genotype from one plate were measured for each replicate. Data are presented as means ± SD, n  =  3. Bars with different lowercase letters are significantly different at *P* < 0.05 (Tukey’s test).
(TIF)

S15 Fig MYB4-GFP fusion did not affect the function of MYB4. (A) Analysis of Cd tolerance in Col, *35S*:*MYB4* and *35S*:*MYB4-GFP* seedlings in the absence or presence of Cd. Three-day-old seedlings grown on 1/2 MS medium were transferred to 1/2 MS medium with or without 50 or 75 μM CdCl_2_ for 2 weeks. Scale bar = 1 cm. (B) Root length of plants described in (A). Three independent experiments were done with similar results, each with three biological replicates. Four plants per genotype from one plate were measured for each replicate. Data are presented as means ± SD, n  =  3. Bars with different lowercase letters are significantly different at *P* < 0.05 (Tukey’s test).
(TIF)

S1 Table Primers used for cloning, RT-PCR, RT-qPCR, ChIP and EMSA assays. (DOCX)
