# Supplementary figures and images for: A MYB4-MAN3-Mannose-MNB1 signaling cascade regulates cadmium tolerance in Arabidopsis
Source: PLoS Genet. 2021 Jun 28;17(6):e1009636. doi: 10.1371/journal.pgen.1009636 (PMC8270467; doi:10.1371/journal.pgen.1009636)

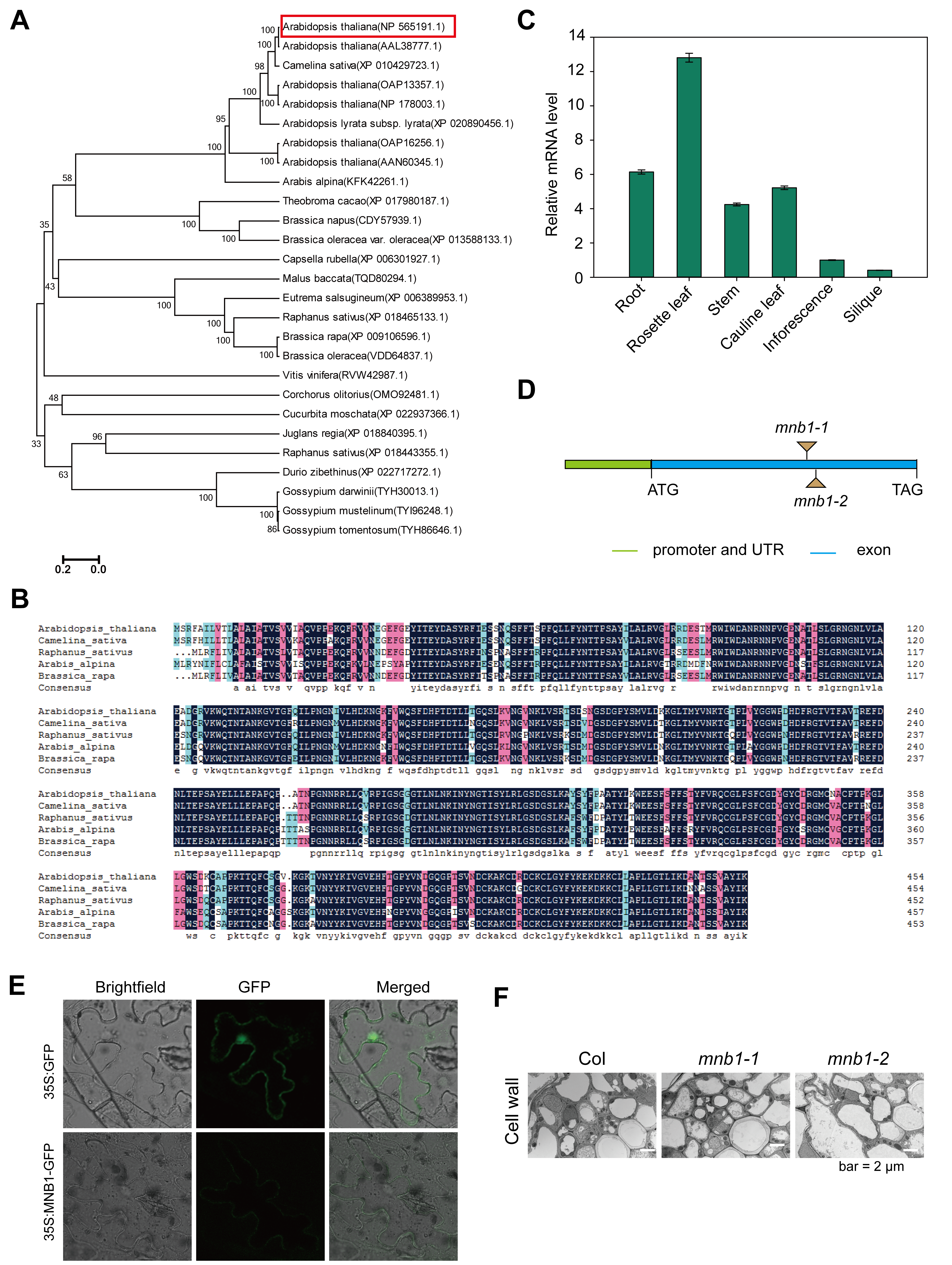

Supplement: S1 Fig — (A) Phylogenic tree of MNB1. (B) Similarity in protein sequences between MNB1 and other proteins. (C) RT-qPCR analysis of MNB1 transcript level in different tissues of wild-type plants. RNA was isolated from roots, rosette leaves, cauline leaves, inflorescence, stem, siliques of the wild-type plants. GAPDH was used as an internal control. Data are presented as means ± SD of three biological replicates. (D) Schematic of T-DNA insertion sites on the locus of MNB1 gene in the mnb1 mutants. (E) Subcellular localization of MNB1. (F) Observation of the cell wall structure of Col and mnb1 mutant by SEM. (TIF) [file pgen.1009636.s001.tif]

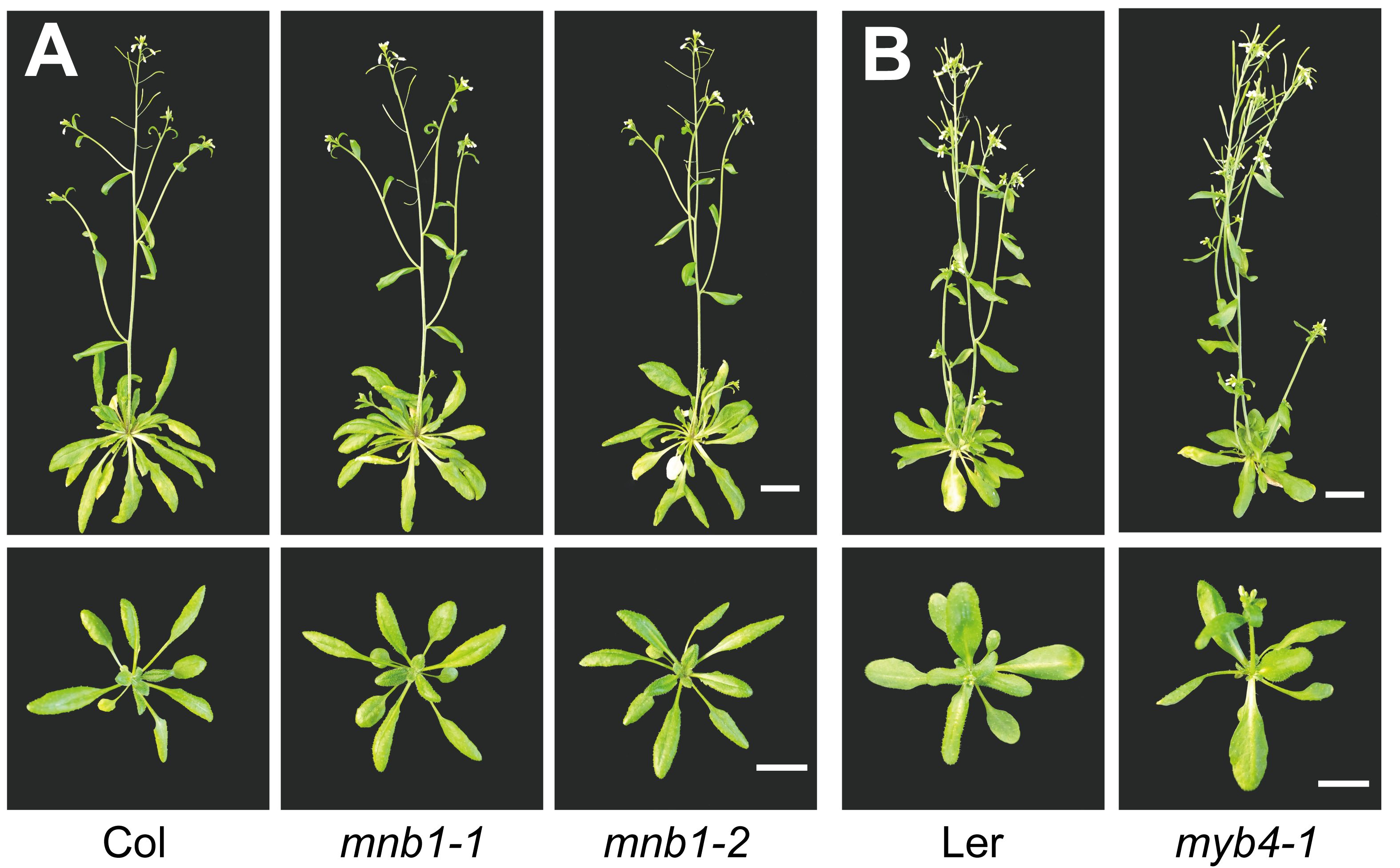

Supplement: S2 Fig — (A) Growth of 6-week-old /4-week-old Col and the mnb1 mutants. (B) Growth of 6-week-old /4-week-old Ler and the myb4-1 mutant. Scale bar = 1 cm. (TIF) [file pgen.1009636.s002.tif]

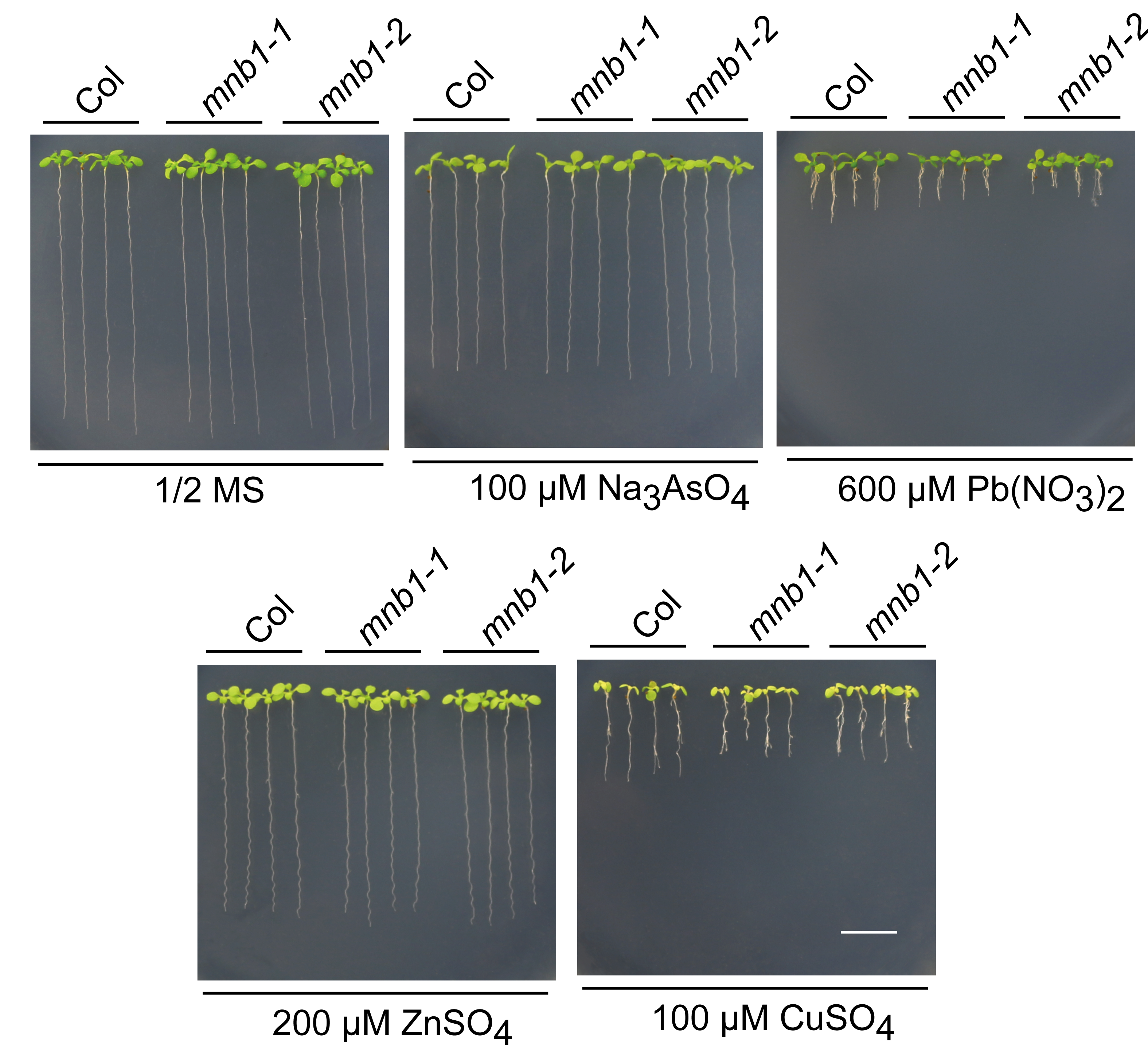

Supplement: S3 Fig — Three-day-old seedlings grown on 1/2 MS medium were transferred to 1/2 MS medium with or without 100 μM Na3AsO4, 600 μM Pb(NO3)2, 200 μM ZnSO4 or 100 μM CuSO4 for about 2 weeks. Scale bar = 1 cm. (TIF) [file pgen.1009636.s003.tif]

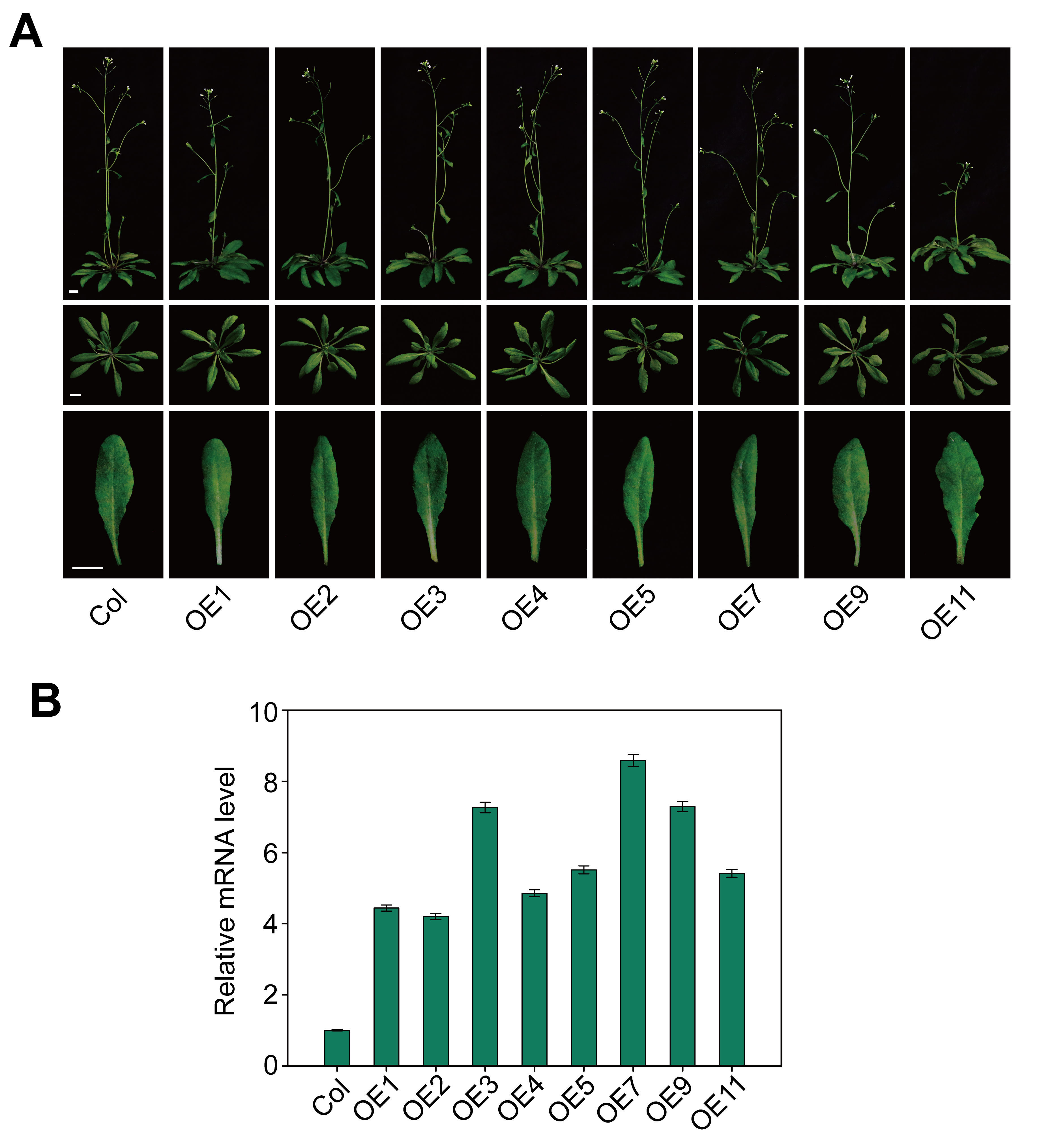

Supplement: S4 Fig — (A) Growth of 6-week-old /4-week-old Col or MNB1-overexpression lines. Scale bar = 1 cm. (B) qRT-PCR analysis of the transcript level of MNB1 in Col and MNB1-overexpression lines. GAPDH was used as an internal control. Data are presented as means ± SD of three biological replicates. (TIF) [file pgen.1009636.s004.tif]

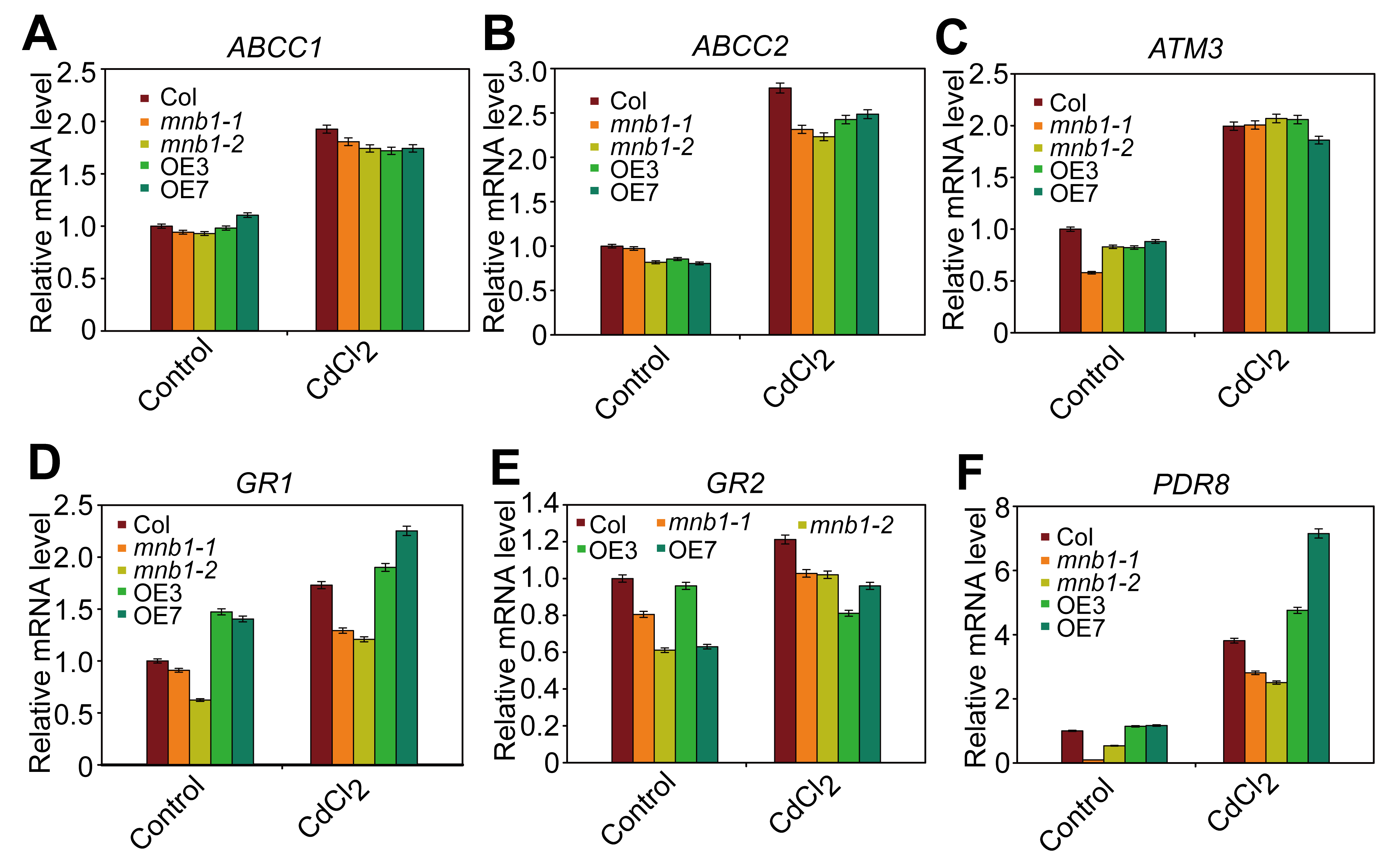

Supplement: S5 Fig — Two-week-old plants grown on 1/2 MS medium were treated with or without 50 μM CdCl2 for 6 h for analysis of transcript levels of genes. GAPDH was used as an internal control. Data are presented as means ± SD, n = 3. (TIF) [file pgen.1009636.s005.tif]

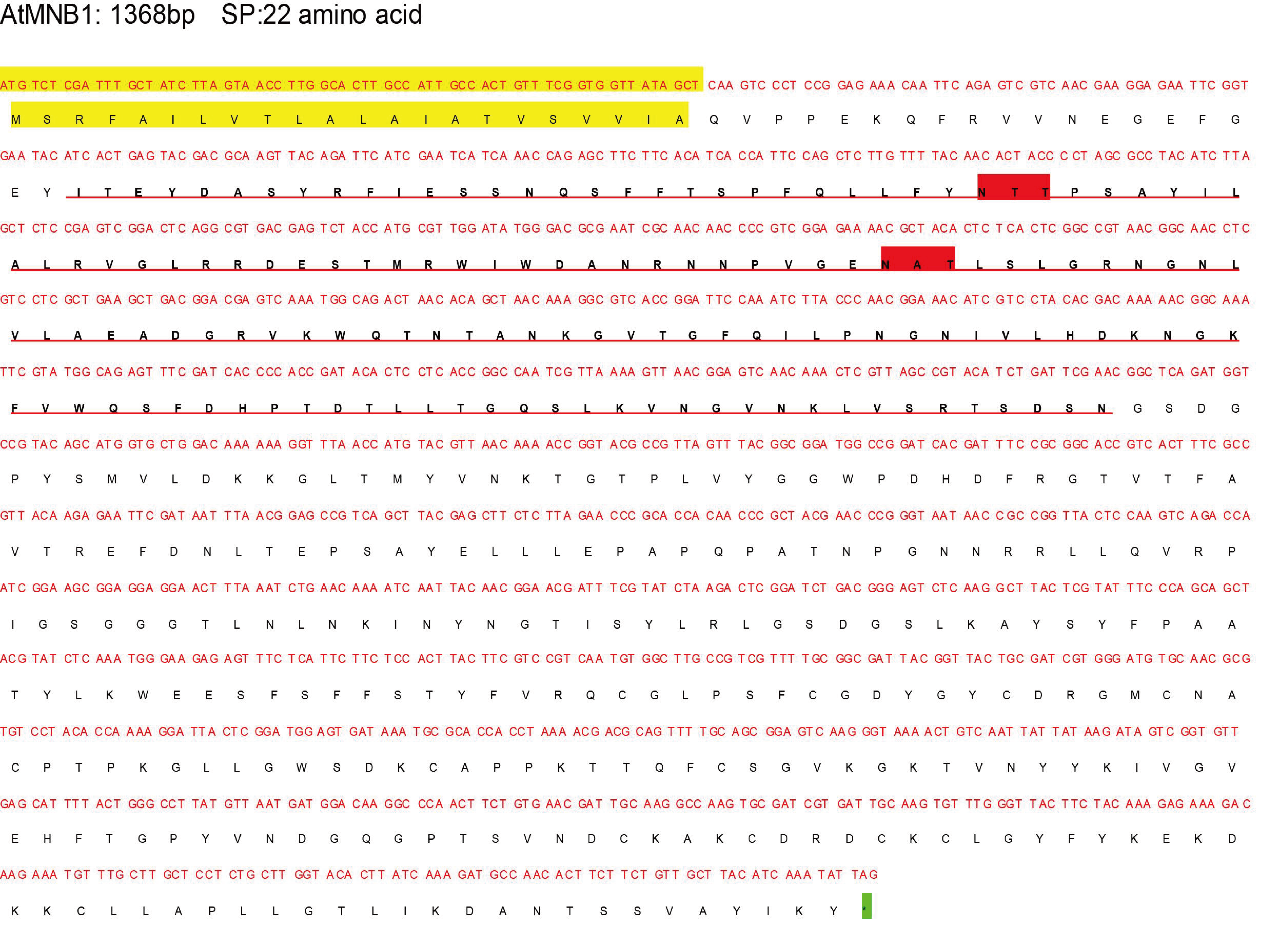

Supplement: S6 Fig — The deduced amino acid sequences by red letters are below the nucleotide sequences by black letters. The signal peptide sequence is on the yellow background. The putative GNA-related lectin is underlined and bold type and the putative N-glycosylation sites are on the red background. The termination codon is marked by an asterisk (*) on a green background. (TIF) [file pgen.1009636.s006.tif]

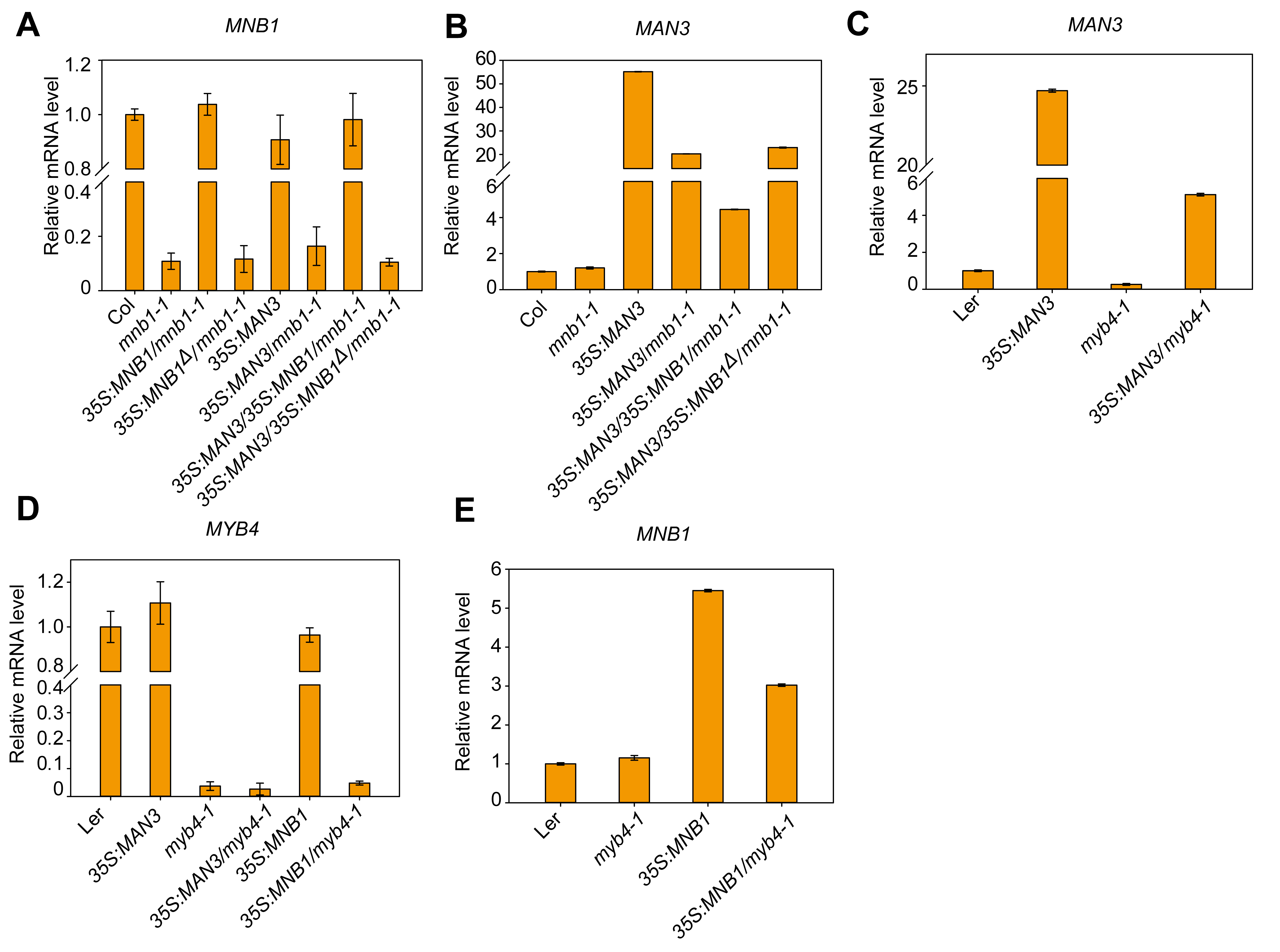

Supplement: S7 Fig — GAPDH was used as an internal control. Data are presented as means ± SD of three biological replicates. (TIF) [file pgen.1009636.s007.tif]

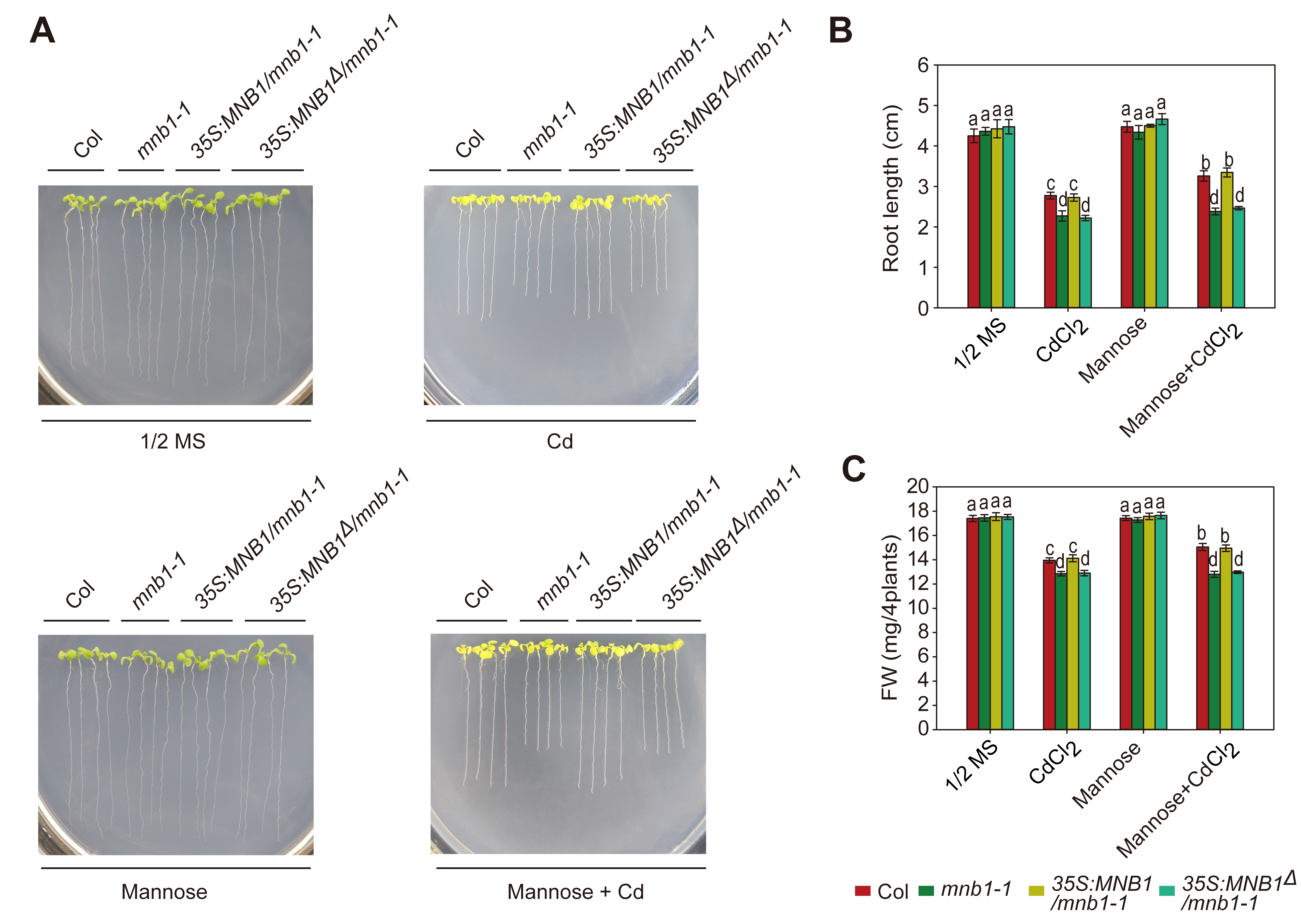

Supplement: S8 Fig — (A) Effect of 1.5 mM mannose treatment on Cd tolerance of the Col, mnb1, 35SMNB1:mnb1-COM and MNB1Δ/mnb1 seedlings with or without 50 μM CdCl2. Three-day-old seedlings grown on 1/2 MS medium were transferred to 1/2 MS medium with or without 50 μM CdCl2 or 1.5 mM mannose for about 2 weeks. Scale bar = 1 cm. (B, C) Root length (B) and fresh weight (C) of plants described in (A). Three independent experiments were done with similar results, each with three biological replicates. Four plants per genotype from one plate were measured for each replicate. Data are presented as means ± SD, n = 3. Bars with different lowercase letters are significantly different at P < 0.05 (Tukey’s test). (TIF) [file pgen.1009636.s008.tif]

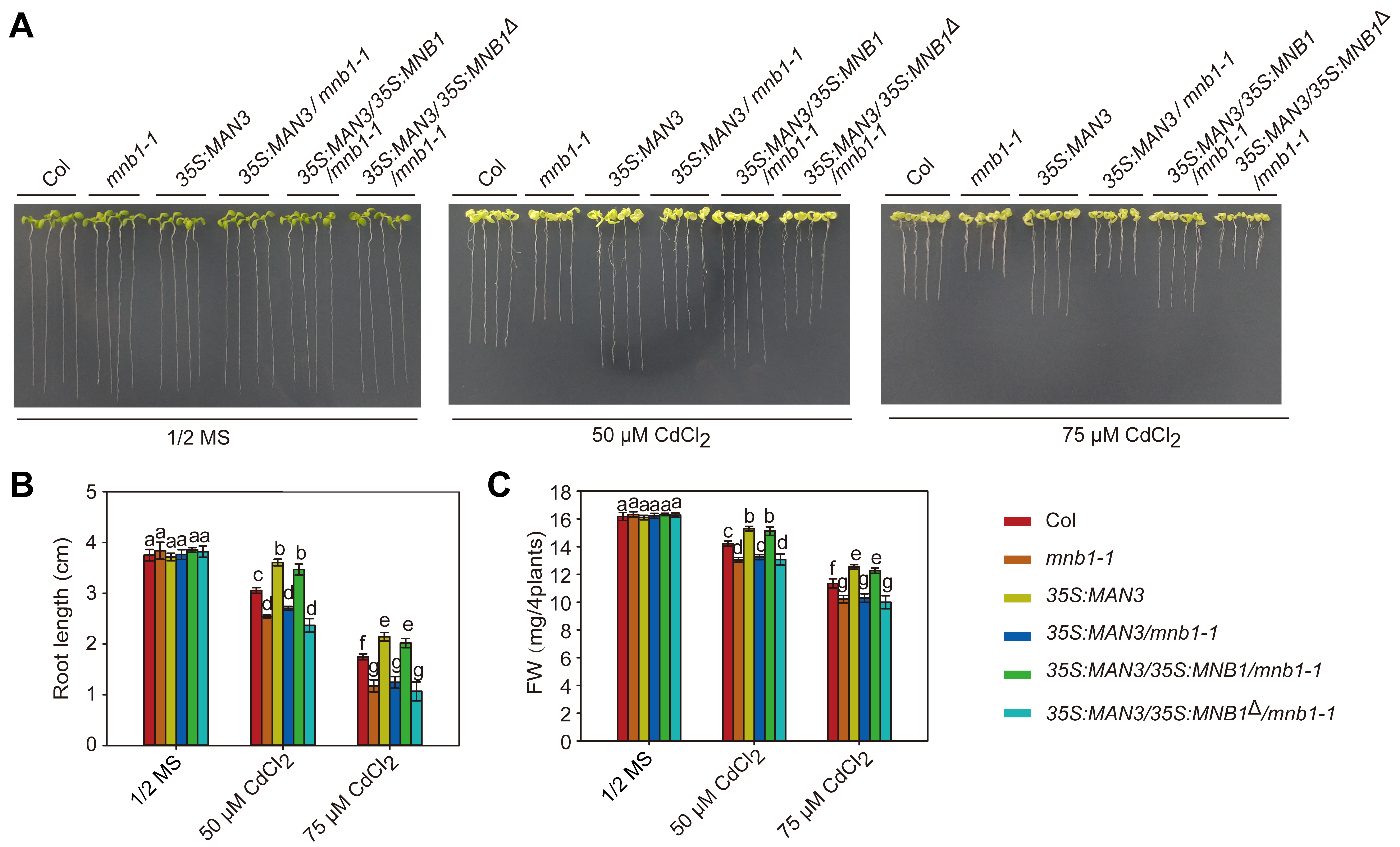

Supplement: S9 Fig — (A) Cd tolerance of the Col, mnb1, MAN3-OE, MAN3-OE/mnbl1, MAN3-OE/35SMNB1:mnb1-COM and MAN3-OE/MNB1Δ/mnb1 seedlings. Three-day-old seedlings grown on 1/2 MS medium were transferred to 1/2 MS medium with or without 50 μM CdCl2 for about 2 weeks. Scale bar = 1 cm. (B, C) Root length (B) and fresh weight (C) of plants described in (A). Three independent experiments were done with similar results, each with three biological replicates. Four plants per genotype from one plate were measured for each replicate. Data are presented as means ± SD, n = 3. Bars with different lowercase letters are significantly different at P < 0.05 (Tukey’s test). (TIF) [file pgen.1009636.s009.tif]

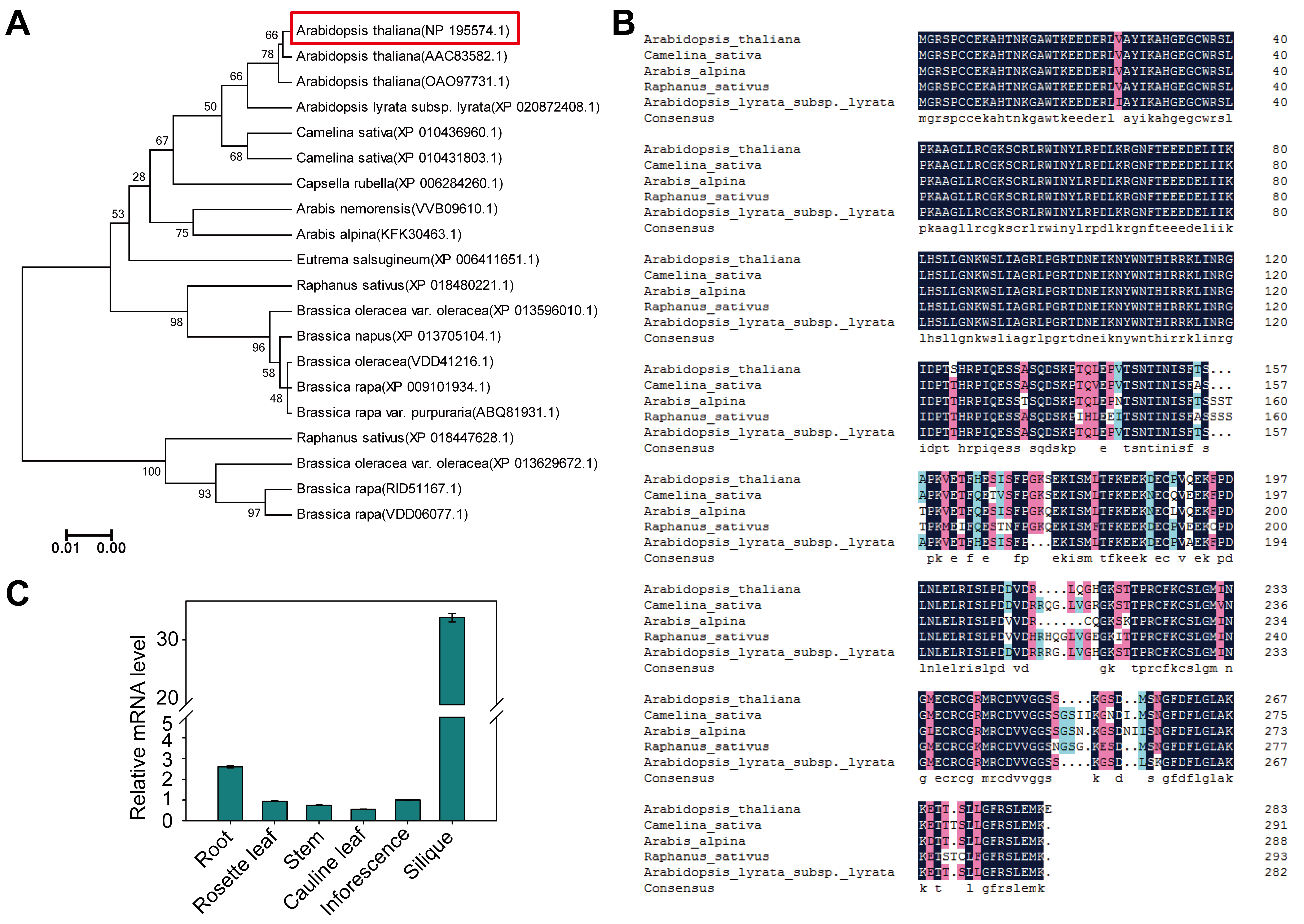

Supplement: S10 Fig — (A) Phylogenic tree of MYB4. (B) Similarity in protein sequences between MYB4 and other proteins. (C) RT-qPCR analysis of MYB4 transcript level in different tissues of wild-type plants. RNA was isolated from roots, rosette leaves, cauline leaves, inflorescence, stem, siliques of the wild-type plants. GAPDH was used as an internal control. Data are presented as means ± SD of three biological replicates. (TIF) [file pgen.1009636.s010.tif]

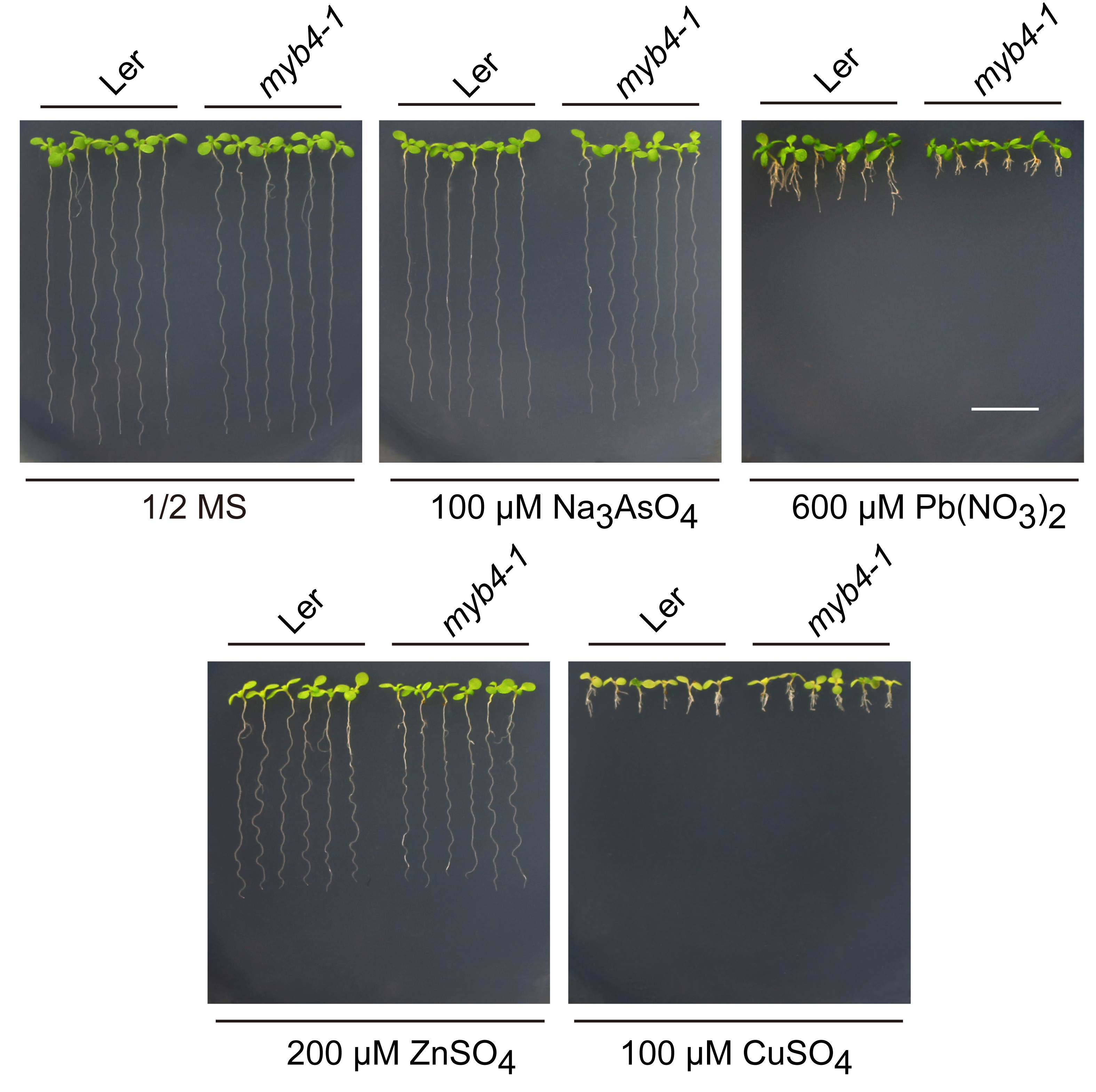

Supplement: S11 Fig — Three-day-old seedlings grown on 1/2 MS medium were transferred to 1/2 MS medium with or without 100 μM Na3AsO4, 600 μM Pb(NO3)2, 200 μM ZnSO4 or 100 μM CuSO4 for about 2 weeks. Scale bar = 1 cm. (TIF) [file pgen.1009636.s011.tif]

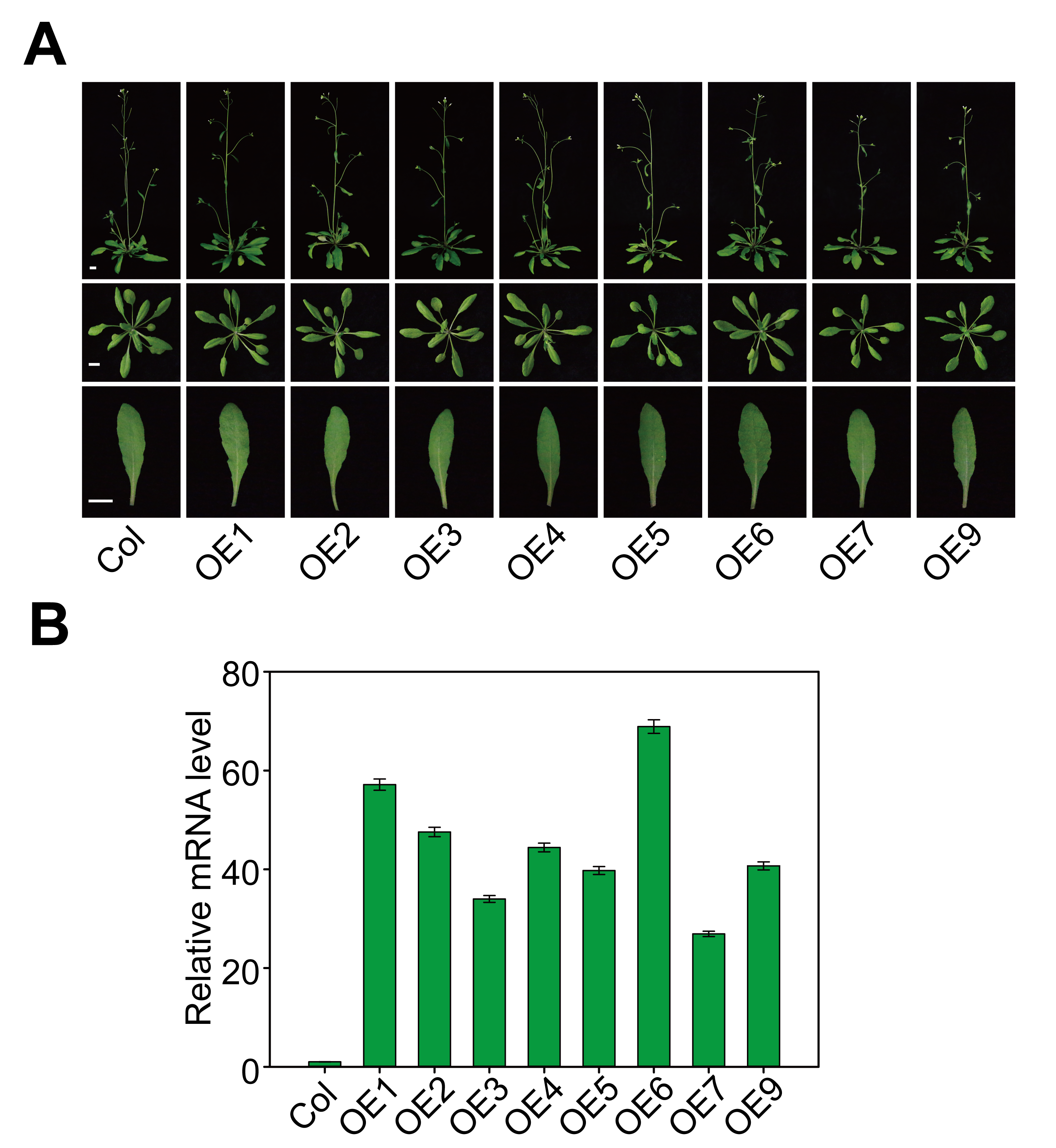

Supplement: S12 Fig — (A) Growth of 6-week-old /4-week-old Col or MYB4-overexpression lines. Scale bar = 1 cm. (B) qRT-PCR analysis of the transcript level of MYB4 in Col and MYB4-overexpression lines. GAPDH was used as an internal control. Data are presented as means ± SD of three biological replicates. (TIF) [file pgen.1009636.s012.tif]

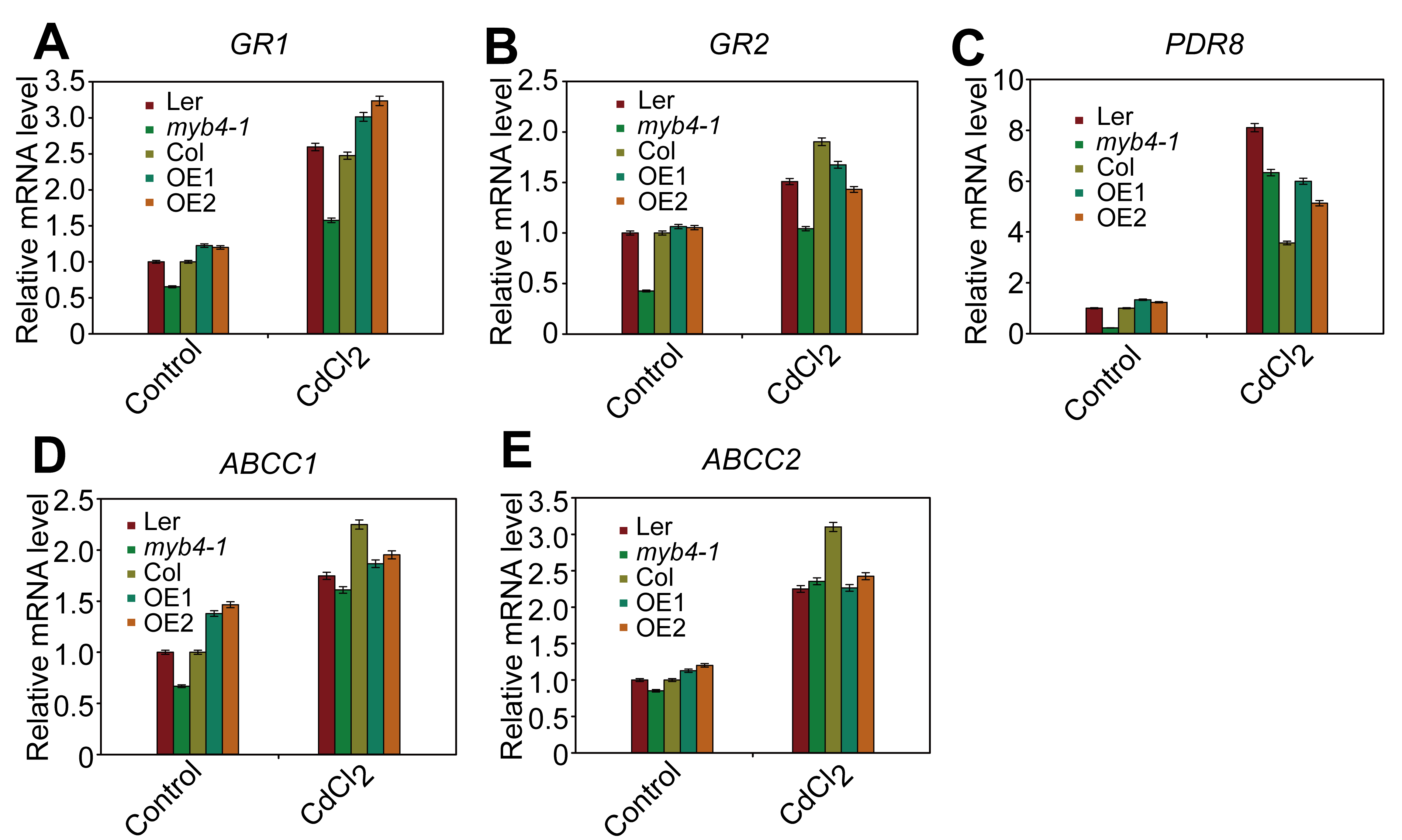

Supplement: S13 Fig — Two-week-old plants grown on 1/2 MS medium were treated with or without 50 μM CdCl2 for 6 h for analysis of transcript levels of genes. GAPDH was used as an internal control. Data are presented as means ± SD, n = 3. (TIF) [file pgen.1009636.s013.tif]

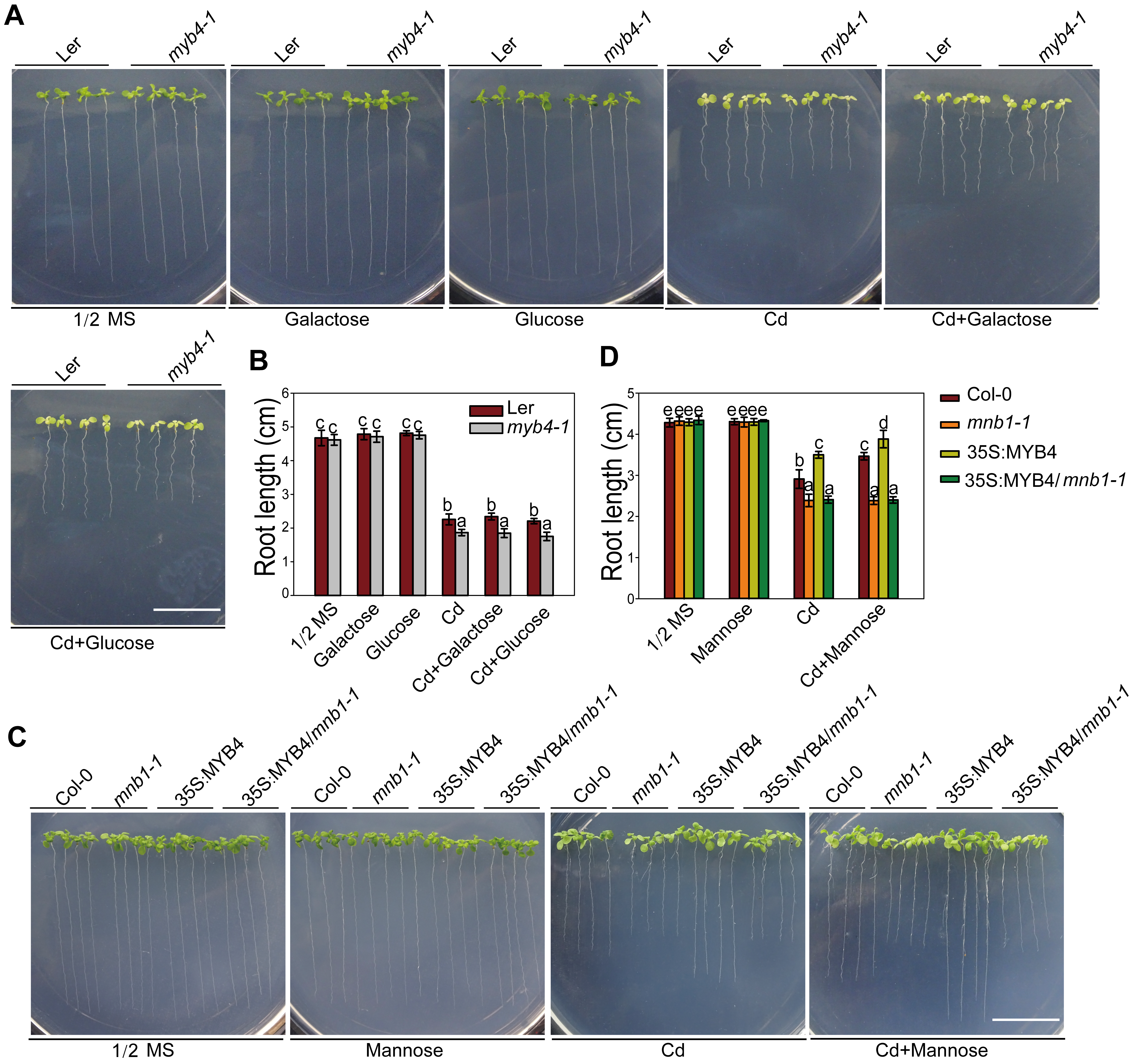

Supplement: S14 Fig — (A) Analysis of Cd tolerance in Ler and myb4-1 mutant seedlings in the absence or presence of Cd, galactose, and glucose. (B) Root length of plants described in (A). (C) Cd and mannose stress phenotypes of the Col, mnb1-1, MYB4-OE, and MYB4-OE/mnb1-1 seedlings. (D) Root length of plants described in (C). In (A) and (C), three-day-old seedlings grown on 1/2 MS medium were transferred to 1/2 MS medium with or without 50 μM CdCl2, 1.5 mM mannose, 1.5 mM galactose, or 1.5 mM glucose, for about 2 weeks. Scale bar = 1 cm. In (B) and (D), three independent experiments were done with similar results, each with three biological replicates. Four plants per genotype from one plate were measured for each replicate. Data are presented as means ± SD, n = 3. Bars with different lowercase letters are significantly different at P < 0.05 (Tukey’s test). (TIF) [file pgen.1009636.s014.tif]

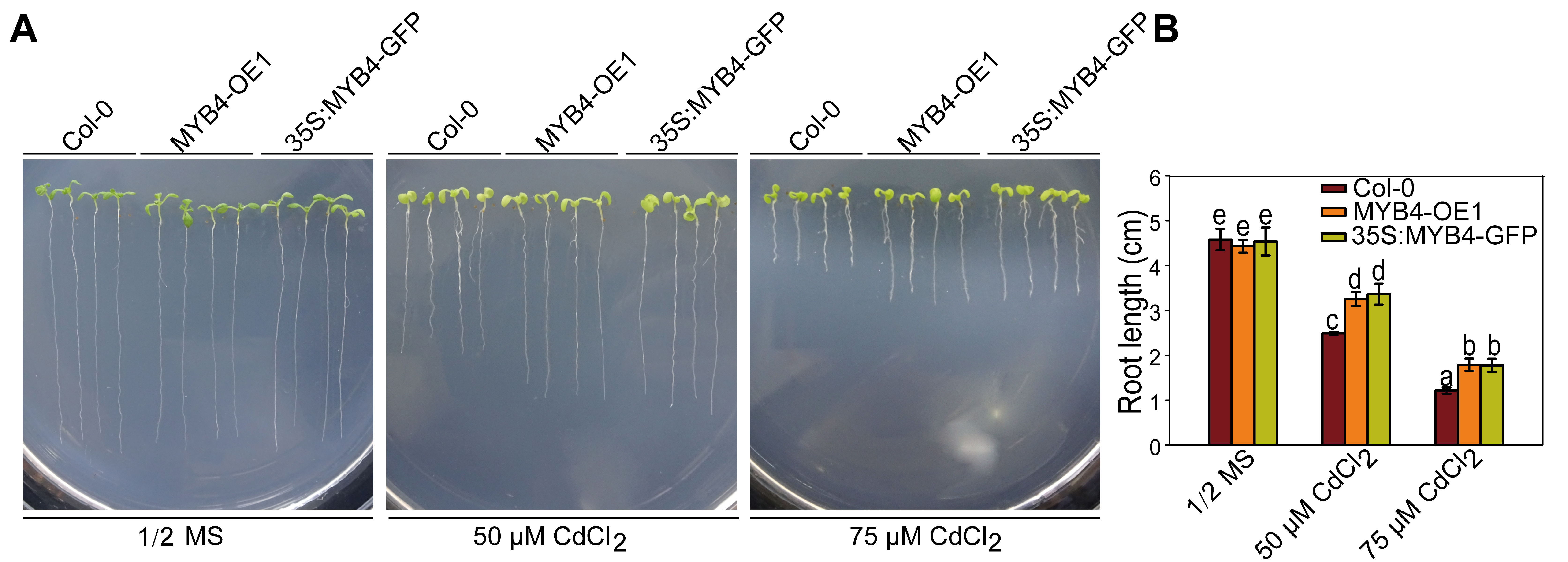

Supplement: S15 Fig — (A) Analysis of Cd tolerance in Col, 35S:MYB4 and 35S:MYB4-GFP seedlings in the absence or presence of Cd. Three-day-old seedlings grown on 1/2 MS medium were transferred to 1/2 MS medium with or without 50 or 75 μM CdCl2 for 2 weeks. Scale bar = 1 cm. (B) Root length of plants described in (A). Three independent experiments were done with similar results, each with three biological replicates. Four plants per genotype from one plate were measured for each replicate. Data are presented as means ± SD, n = 3. Bars with different lowercase letters are significantly different at P < 0.05 (Tukey’s test). (TIF) [file pgen.1009636.s015.tif]
