# Supplementary material for: A MYB4-MAN3-Mannose-MNB1 signaling cascade regulates cadmium tolerance in Arabidopsis
Source: PLoS Genet. 2021 Jun 28;17(6):e1009636. doi: 10.1371/journal.pgen.1009636 (PMC8270467; doi:10.1371/journal.pgen.1009636)
Supplement: S1 Table — (DOCX) [file pgen.1009636.s016.docx]

**S1Table. Primers used for cloning, RT-PCR, RT-qPCR, ChIP and EMSA assays**

| Primer Name Primer Sequences (5’-3’) |
| --- |
| **For cloning and genotyping**  mnb1-1-F TGAGTCCCAACGACCATCTAC  mnb1-1-R ATTCCAAATCTTACCCAACGG  mnb1-2-F TGAGTCCCAACGACCATCTAC  mnb1-2-R ATTCCAAATCTTACCCAACGG  MNB1OE-S CGG**GGTACC**ATGTCTCGATTTGCTATCTTAGTAA  MNB1OE-AS CCG**CTCGAG**CTAATATTTGATGTAAGCAACAGAA  MNB1GFP-S CGG**GGTACC**ATGTCTCGATTTGCTATCTTAGT  MNB1GFP-AS CCG**CTCGAG**ATATTTGATGTAAGCAACAGAA  mnb1-COM-S1 CGG**GGTACC**ATAGTGTATGTTCTCCGCTCA  mnb1-COM-AS2 CCG**CTCGAG**AATTTCTCGATTTTACCGTCT  mnb1Δ-AS1 GTACGGACCATCTGAGCCGATGTATTCACCGAATTCTCC  mnb1Δ-S2 GGAGAATTCGGTGAATACATCGGCTCAGATGGTCCGTAC  MYB4OE-S CGG**GGTACC**ATGGGAAGGTCACCGTGCT  MYB4OE-AS CCG**CTCGAG**TTATTTCATCTCCAAGCTTCGAAAG  MYB4GFP-S CGG**GGTACC**ATGGGAAGGTCACCGTGCT  MYB4GFP-AS CCG**CTCGAG**TTTCATCTCCAAGCTTCGAAAG  MAN3OE-S CGG**GGTACC**ATGAAGTGTTTGTGTTTTGTCG  MAN3OE-AS CCG**CTCGAG**TTAAATTTTAGTTTTTGATAAC  MAN3pro-F CGG**GGTACC**TTGAAGACTGAGATAAGTAAACACCC  MAN3pro-R CCG**CTCGAG**CAAGAAAGCTTGTGACTTTGGCTC  GSH1pro-F CCG**GAATTC**GCACGTGTCCACCTACCTTACC  GSH1pro-R TGC**TCTAGA**GAGCGCCATGGTATATATAGCTCC  GSH2pro-F TGC**TCTAGA**ATTGTGGACAAAGAGTGTCAACAG  GSH2pro-R CCC**AAGCTT**CTCAGTCAAACAAAGATCGCTAC  PCS1pro-F CCG**CTCGAG**ATGTAGGGAATCTGAGAACCTCTTG  PCS1pro-R CCG**CTCGAG**ATGTAGGGAATCTGAGAACCTCTTG  PCS2pro-F CCG**CTCGAG**AAAATCGTCGAGAGAGCGTTC  PCS2pro-R CCC**AAGCTT**TGTCAGAGTTTGACTATGGAGCA  **For RT-qPCR**  GAPDH-qS TTGGTGACAACAGGTCAAGCA  GAPDH-qAS AAACTTGTCGCTCAATGCAATC  MNB1-qS AAGATAGTCGGTGTTGAGCATT  MNB1-qAS AAGTGTACCAAGCAGAGGAGC  MYB4-qS CAAGGGCATGGAAAGTCAAC  MYB4-qAS TTGCTGCTACCTCCGACTAC  GSH1-qS GATGGTTTAGAGCGCAGAGG  GSH1-qAS TACGCTTTGTCCCCATTCTC  GSH2-qS ACCAACTGCATTCCCAGAAG  GSH2-qAS GCCATCCAAGCTAACACGAT  PCS1-qS TCAGGGATCAAAGACCAAGC  PCS1-qAS CCGTCGAAGATGCAATACCT  PCS2-qS ATCCTCCTCACTGGGTTCCT  PCS2-qAS GGTTCTCTGTGGGGTCTTGA  GR1-qS AGAATTTCCCAAGCGTGCTA  GR1-qAS AGTGCCCTCATTTCGTCATC  GR2-qS TCCCAATCGCAATTCATCTAC  GR2-qAS TGGTAATAGCGGTGGTTTGAG  ABCC1-qS GTTGACTGCGTCATTAGCCG  ABCC1-qAS AACTGAGAAGCAAACCCATCG  ABCC2-qS ATTTCAGCGTGGGACAGAGG  ABCC2-qAS ATTGAGACGGTGAGCGATA  ATM3-qS GACATCACAAATACAAGTGACGC  ATM3-qAS CACTATTCCAATTTGATAGCTGC  PDR8-qS CTACACTCTTCCTGAGAACCGAA  PDR8-qAS TCATAGCCATCTCCGCAAACCC  **For RT-PCR**  ACTIN8-sqS GAGACAACTTACAACTCGATC  ACTIN8-sqAS CTGTGGACAATGCCTGGAC |
| MYB4-sqS CACTTCTGCTCCAAAGGTCG  MYB4-sqAS CCATTGCTCATGTCACTCCC  **For ChIP-qPCR and Y1H**  MAN3-P1F GGAAGATAATCAACATGTTGACCACA  MAN3-P1R CGAACATTCTAAGTCGTGGATTTTC  MAN3-P2F GGCATCGAGGGAAACAAATCAT  MAN3-P2R TCTTCCTAATCAATACTGGCTAACCA  MAN3-P3F CGAGCATTGTTTTGGGAGGTTATTA  MAN3-P3R GCTAAACTTTACTTTCTTGTGGTCTC  MAN3-P4F TCCTTGTTGAACACCTCAGCC  MAN3-P4R GTCCAACCTTTGAAACCTGTGC  MAN3-H1Y-1F GG**GGTACC**CGAGCATTGTTTTGGGAGGTTATTA  MAN3-H1Y-1R CCG**CTCGAG**GCTAAACTTTACTTTCTTGTGGTCTC  MAN3-H1Y-2F GG**GGTACC**GGCATCGAGGGAAACAAATCAT  MAN3-H1Y-2R CCG**CTCGAG**TCTTCCTAATCAATACTGGCTAACCA  MAN3-H1Y-3F GG**GGTACC**TCCTTGTTGAACACCTCAGCC  MAN3-H1Y-3R CCG**CTCGAG**GTCCAACCTTTGAAACCTGTGC  MAN3-H1Y-4F GG**GGTACC**GGAAGATAATCAACATGTTGACCACA  MAN3-H1Y-4R CCG**CTCGAG**CGAACATTCTAAGTCGTGGATTTTC  **For protein expression**  His-mnb1-1F CG**GGATCC**GATGTCTCGATTTGCTATC  His-mnb1-1R CC**CTCGAG**ATATTTGATGTAAGCAACAG  His-mnb1-2F CG**GGATCC**GATGCAAGTCCCTCCGG  His-mnb1-2R CC**CTCGAG**ATATTTGATGTAAGC AACAGA  His-mnb1-3F CG**GGATCC**GATGTACTCACTGAGTACGA  His-mnb1-3R CC**CTCGAG**TCGAATCAGATGTACGGC  His-mnb1-4F CG**GGATCC**GATGGGCTCAGATGGTCC  His-mnb1-4R CC**CTCGAG**ATATTTGATGTAAGCAACAGAAG  **Oligonucleotides used for EMSA assay**  EMSA-Bio-p1F: GGTTTACAAAT**CAACAACC**ACTGAACTTTT  EMSA-Bio-p1R: AAAAGTTCAGTGGTTGTTGATTTGTAAACC  EMSA-Bio-P2F: AAGATGGGTTT**GCTTGGTG**AGACCACAAGA  EMSA-Bio-P2R: TCTTGTGGTCTCACCAAGCAAACCCATCTT  EMSA-Bio-P3F: CAAATCATATA**GTTTGGTG**ACTGATTTCAA  EMSA-Bio-P3R: TTGAAATCAGTCACCAAACTATATGATTTG  EMSA-Bio-P4F: AGTTAGTCAATG**AACGG**TTTTCAAATAAAG  EMSA-Bio-P4R: CTTTATTTGAAAACCGTTCATTGACTAACT  EMSA-Mut-P1F: GGTTTACAAAT**CGACGAGC**ACTGAACTTTT  EMSA- Mut-P1R: AAAAGTTCAGTGGTTGTTGATTTGTAAACC  EMSA- Mut-P2F: AAGATGGGTTT**CCAGGGTC**AGACCACAAGA  EMSA- Mut-P2R: TCTTGTGGTCTCACCAAGCAAACCCATCTT  EMSA- Mut-P3F: CAAATCATATA**GAGTGCTC**ACTGATTTCAA  EMSA- Mut-P3R: TTGAAATCAGTCACCAAACTATATGATTTG  EMSA- Mut-P4F: AGTTAGTCAATG**GCCAG**TTTTCAAATAAAG  EMSA- Mut-P4R: CTTTATTTGAAAACCGTTCATTGACTAACT |

**Accession numbers**

Sequence data from this article can be found in the Arabidopsis Genome Initiative or GeneBank/EMBL database under the following accession numbers: *MNB1* (*AT1g78830*), *MYB4* (*AT4g38620*), *GSH1* (*At4g23100*), *GSH2 (AT5G27380), PCS1 (AT5G44070), PCS2 (AT1G03980), GR1 (AT3G24170), GR2* (*AT3G54660*)*, ABCC1 (AT1G30400), ABCC2 (AT2G34660), PDR8 (At1g59870), ATM3* (*At5g58270*), *GAPDH (AT1G13440)*, and *ACTIN8 (AT1G49240).*
